# APOE genotype dependent molecular abnormalities in the cerebrovasculature of Alzheimer’s disease and age-matched non-demented brains

**DOI:** 10.1186/s13041-021-00803-9

**Published:** 2021-07-08

**Authors:** Joseph O. Ojo, Jon M. Reed, Gogce Crynen, Prashanthi Vallabhaneni, James Evans, Benjamin Shackleton, Maximillian Eisenbaum, Charis Ringland, Anastasia Edsell, Michael Mullan, Fiona Crawford, Corbin Bachmeier

**Affiliations:** 1 Department of Experimental Neuropathology, Roskamp Institute, Sarasota, FL 34243 USA; 2 James A. Haley Veterans’ Hospital, Tampa, FL USA; 3 The Open University, Milton Keynes, UK; 4 Boehringer Ingelheim Pharmaceuticals, Inc., Ridgefield, CT USA; 5 Bay Pines VA Healthcare System, Bay Pines, FL USA

**Keywords:** Cerebrovasculature, APOE, Aging, Alzheimer’s disease, Mural cells, Endothelial cells, Proteomics, Mass spectrometry

## Abstract

Cerebrovascular dysfunction is a hallmark feature of Alzheimer's disease (AD). One of the greatest risk factors for AD is the apolipoprotein E4 (E4) allele. The APOE4 genotype has been shown to negatively impact vascular amyloid clearance, however, its direct influence on the molecular integrity of the cerebrovasculature compared to other APOE variants (APOE2 and APOE3) has been largely unexplored. To address this, we employed a 10-plex tandem isobaric mass tag approach in combination with an ultra-high pressure liquid chromatography MS/MS (Q-Exactive) method, to interrogate unbiased proteomic changes in cerebrovessels from AD and healthy control brains with different APOE genotypes. We first interrogated changes between healthy control cases to identify underlying genotype specific effects in cerebrovessels. EIF2 signaling, regulation of eIF4 and 70S6K signaling and mTOR signaling were the top significantly altered pathways in E4/E4 compared to E3/E3 cases. Oxidative phosphorylation, EIF2 signaling and mitochondrial dysfunction were the top significant pathways in E2E2 vs E3/E3cases. We also identified AD-dependent changes and their interactions with APOE genotype and found the highest number of significant proteins from this interaction was observed in the E3/E4 (192) and E4/E4 (189) cases. As above, EIF2, mTOR signaling and eIF4 and 70S6K signaling were the top three significantly altered pathways in E4 allele carriers (i.e. E3/E4 and E4/E4 genotypes). Of all the cerebrovascular cell-type specific markers identified in our proteomic analyses, endothelial cell, astrocyte, and smooth muscle cell specific protein markers were significantly altered in E3/E4 cases, while endothelial cells and astrocyte specific protein markers were altered in E4/E4 cases. These proteomic changes provide novel insights into the longstanding link between APOE4 and cerebrovascular dysfunction, implicating a role for impaired autophagy, ER stress, and mitochondrial bioenergetics. These APOE4 dependent changes we identified could provide novel cerebrovascular targets for developing disease modifying strategies to mitigate the effects of APOE4 genotype on AD pathogenesis.

**Supplementary Information:**

The online version contains supplementary material available at 10.1186/s13041-021-00803-9.

## Introduction

Alzheimer’s disease (AD) is the most predominant type of dementia, which to date remains untreatable. One of the most common preclinical features of AD is vascular dysfunction, evident by deficits in cortical blood flow and metabolic activity, many years before the onset of neurological symptoms [1–5]. At autopsy, vascular lesions are routinely reported in early prodromal and later stages of AD. This is typified by cerebral amyloid angiopathy (CAA) [6], lesions of cerebral small vessel disease (CSVD) such as lacunar infarcts and microhemorrhages [7–9], cerebral atherosclerosis [10–12], degenerating small blood vessels, capillaries and mural cells [13–17], weakened blood–brain barrier (BBB) [18], and buildup of phagolysosomes-lipofuscin deposits and dysmorphic mitochondria in cerebrovascular cells [3, 16, 19]. Some of these vascular lesions can act as protagonists negatively modifying the clinical presentation of AD [7–9, 20–22].

It remains unknown whether these vascular changes in AD are a prelude to, or a direct consequence of A $\beta$ toxicity [23]. Early vascular dysfunction (pre-amyloidosis) can contribute to an increase in Aβ accumulation in leptomeninges and along cerebral blood vessels as perivascular drainage along these sites is a preferred route of amyloid clearance from the brain [23–25]. Deposition of A $\beta$ on vessel walls can also induce cerebrovascular changes that further impairs vascular hemodynamics [26, 27], increasing cerebral blood pressure and reducing cerebral perfusion [28, 29]. The specific molecular triggers and factors driving these degenerative cerebrovascular phenotypes and the timing of these events in the sequelae of AD remain elusive.

One of the strongest risk factors for AD is the ε4 allele of the apolipoprotein E (APOE4) genotype [30–32]. The APOE protein is a multifunctional protein which contains three distinct functional domains, an N-terminal receptor binding domain, a random coil region, and the C-terminal lipid-binding domain. The different isoforms of APOE (E2, E3, E4) differ by a single amino acid change which impacts their lipid binding capabilities. In the brain, APOE is produced and secreted primarily by astrocytes and microglia and it is subsequently lipidated to form nascent high-density lipoprotein (HDL)-like particles. This lipidated form of APOE plays a vital role in cholesterol transport, lipid metabolism [33], and Aβ metabolism and clearance [30, 34].

Some of the early clues linking APOE with vascular pathobiology involved early antibody trials showing a link between the APOE4 allele and recurrent hemorrhages in cerebral amyloid angiopathy (CAA) patients [35–37]. Since then postmortem studies have revealed a correlation between APOE4 and vascular lesions such as CAA [38, 39], CSVD [40–42], atherosclerosis [43, 44] and deficits in cerebral blood flow [45]. More recent work has confirmed that the APOE4 allele accelerates BBB breakdown in elderly unimpaired individuals and, to a greater extent, in demented patients [42, 46–49]. Preclinical models expressing human APOE isoforms, have also shown that the E4 allele drives early pericyte degeneration and BBB abnormalities prior to neuronal dysfunction [42]. Thus it appears that APOE may significantly contribute to the degenerative cerebrovascular phenotypes in AD. Yet the specific details of how APOE isoform variants influence the molecular integrity of the cerebrovasculature in normal and pathogenic (AD) settings remains elusive.

To address this, we will use our state-of-the-art unbiased proteomic (mass spectrometry) based platform to conduct a detailed characterization and assessment of molecular changes in protein expression levels, molecular pathways and biofunctions significantly altered in the cerebrovessels of AD patients compared to healthy control cases from different APOE genotype backgrounds (E2/E2, E2/E3, E3/E3, E3/E4 and E4/E4). Unbiased proteomic analysis is an extremely powerful tool which can provide a very expansive interrogation of the molecular response in neurodegenerative diseases [50–53] and can lead to the identification of pathogenic mechanisms and novel molecular targets [54–56] for therapeutic exploration. Herein, we have utilized a novel protein extraction protocol, separating isolated cerebrovessels into cytosolic, membrane and nuclear fractions to increase the depth of the protein mining process, and coupled this with a 10-plex tandem isobaric mass tag (TMT) approach for interrogation with an ultra-high pressure liquid chromatography MS/MS (Q-Exactive) method [57]. In this study, we have detailed the unique molecular profiles and pathogenic mechanisms driven by APOE variant genotypes in cerebrovessels of the inferior frontal gyrus of AD and control patients.

## Methods

### Human brain tissue and patient demographics

Human brain tissue from the inferior frontal gyrus were provided mainly from Dr. Thomas Beach, Director of the Brain and Body Donation Program at Sun Health Research Institute (Sun City, AZ) in accordance with the institutional bioethics guidelines. Additional samples were requested from the NIH BrainBank repository (University of Maryland and Ican school of medicine, Mount Sinai, NY). Autopsies were conducted within 4–5 h after death from non-demented control subjects with no history of AD diagnosis, and AD subjects. Neuropathological post-mortem diagnosis of AD was determined using the Consortium to Establish a Registry for Alzheimer's Disease (CERAD) diagnostic criteria and the consensus recommendation by the National Institute for Aging/Reagan Institute Working Group. Braak staging was used to characterize the neurofibrillary tangle (NFT) distribution. The severity of CAA was performed according to Vonsattel et al. [58], and the stage of topographical expansion of CAA was assessed as previously described by Thal et al. [27] based on a 4 point numerical conversion per region. Global scores for amyloid, tangle and CAA burden from the microscopic lesion densities were calculated based on the sums of the scores from all regions interrogated. A summary of patient demographics, clinical information and APOE genotype background of brain donors used in the study is provided in Tables 1, 2.

**Table 1:** List of control and Alzheimer’s disease cases, their demographics, APOE genotype, and randomization of samples for Tandem Mass Tag isobaric 10-plex multiplexing

DX	APOE genotype	Mean age [years]	Male	Female	N per group	TMT 10 Plex labels
Control	E2/E2	71.5 ± 8.58	33.3% (2/6)	66.7% (4/6)	6	TMT − 126
Control	E2/E3	80 ± 5.85	50% (3/6)	50% (3/6)	6	TMT -127N
Control	E3/E3	83 ± 3.04	100% (6/6)	0% (0/6)	6	TMT -127C* (denominator)
Control	E3/E4	84.17 ± 3.64	60% (3/5)	40% (2/5)	5	TMT -128N
Control	E4/E4	45.83 ± 4.39	66.7% (4/6)	33.3% (2/6)	6	TMT -128C
AD	E2/E2	90 ± 17.0*	0% (0/3)	100% (3/3)	3	TMT -129N
AD	E2/E3	89 ± 4.14	66.7% (4/6)	33.3% (2/6)	6	TMT -129C
AD	E3/E3	87 ± 3.27	16.67% (1/6)	83% (5/6)	6	TMT -130N
AD	E3/E4	78.5 ± 3.60	50% (3/6)	50% (3/6)	6	TMT -130C
AD	E4/E4	84.8 ± 4.55	40% (2/5)	60% (3/5)	5	TMT -131
Total Control	All Genotype	72.90 ± 3.49	37.9% (18/29)	62.1% (11/29)	29	-126, -127N, -127C, -128N, -128C
Total AD	All Genotype	85.24 ± 2.10	38.5% (10/26)	61.5% (16/26)	26	-129N, -129C, -130N, -130C, -131

**Table 2:** Shows the clinical background of patients focusing on brain weight and scores for the mini-mental state examination (MMSE), Amyloid plaque, Tangle, Braak staging, White matter and CAA

DX	APOE genotype	Brain weight	MMSE score	Amyloid plaque score	Tangle score	Braak score	White matter score	CAA score
Control	E2/E2	1207.33 ± 45.8	29 (from 1 case)	3.63 ± 2.4	3.25 ± 1.8	2.75 ± 0.3	2.5 ± 1.5	4 ± 4 (50%)
Control	E2/E3.	1286 ± 48.8	28.6 ± 0.94	1.66 ± 1.1	3.54 ± 0.6	2.17 ± 0.4	2.5 ± 0.7	0 (0%)
Control	E3/E3	1222 ± 34.7	28 ± 0.96	1.33 ± 1.3	2.67 ± 0.79	2.17 ± 0.4	1.67 ± 0.6	2.83 ± 1.9 (40%)
Control	E3/E4	1193.75 ± 82.4	28.25 ± 0.3	3.75 ± 2.4	4.21 ± 0.9	3 ± 0.3	3.25 ± 1.9	0.4 ± 0.4 (20%)
Control	E4/E4	1310 ± 40	N/A	N/A	N/A	N/A	N/A	N/A
AD	E2/E2	N/A	N/A	7.83 ± 2.4	15 (from 1 case)	5.67 ± 0.3	6 (from 1 case)	12 (from 1 case)
AD	E2/E3	1104.83 ± 80.9	18.3 ± 3.0	9.3 ± 1.7	8 ± 1.1	4.16 ± 0.2	3.3 ± 0.8	4.17 ± 1.7 (60%)
AD	E3/E3	1060 ± 37.9	6.67 ± 5.7	13.04 ± 0.97	10.4 ± 2.12	4.67 ± 0.61	5.83 ± 1.9	3.4 ± 1.3 (66.6%)
AD	E3/E4	1036.8 ± 49.3	18 ± 3.2	13 ± 0.6	13 ± 0.9	5.167 ± 0.3	2.8 ± 1.3	4.6 ± 0.9 (100%)
AD	E4/E4	959 ± 18.1	11 ± 2	14.25 ± 0.31	14.5 ± 0.32	6 ± 0	6.2 ± 0.9	8.33 ± 1.4 (100%)

### Isolation of enriched cerebrovessel fractions

**Fig. 1:**
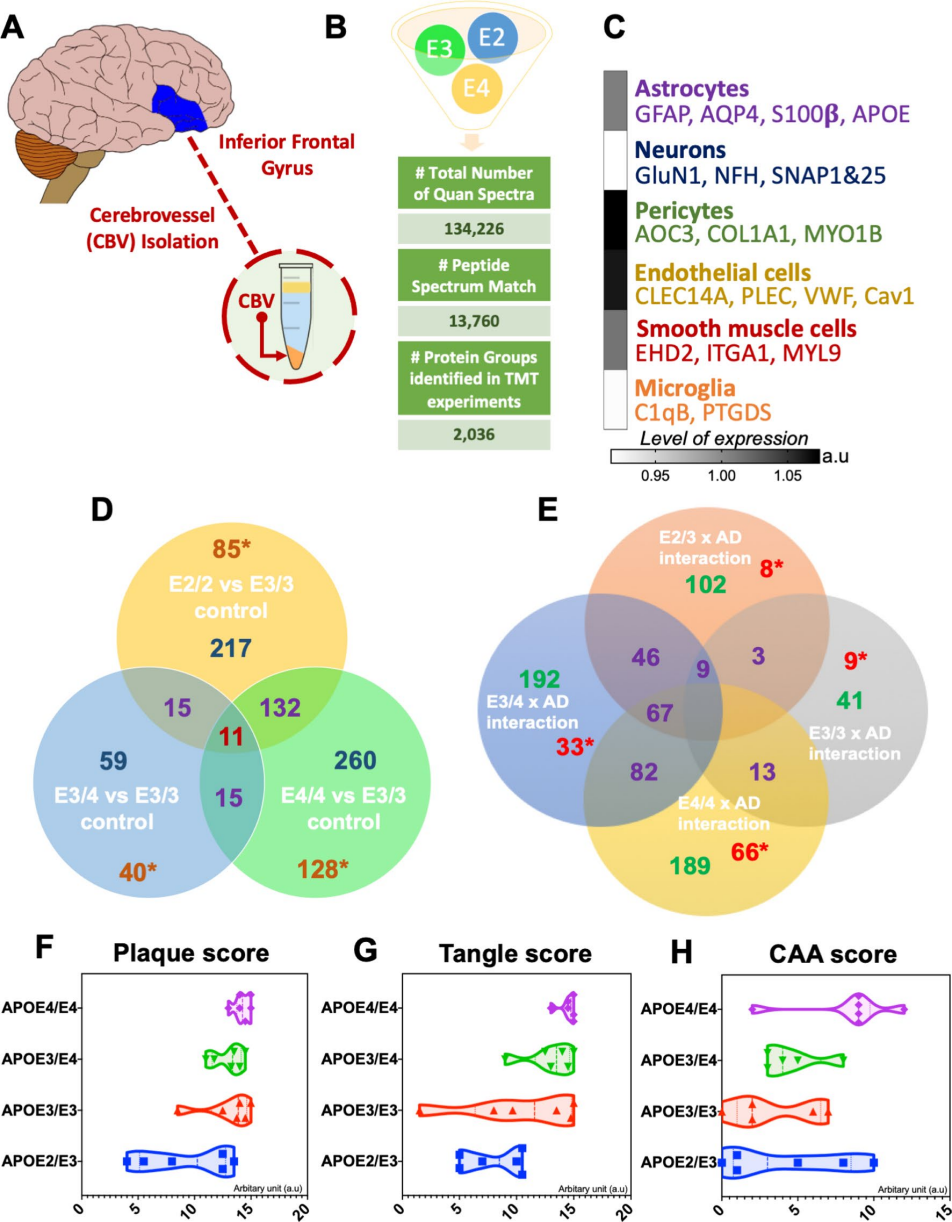
Summary of liquid chromatography/mass spectrometry (LC/MS) and proteomic analyses of tissue from the cerebrovasculature isolated from the inferior frontal gyrus in Alzheimer’s disease (AD) and healthy control cases from different APOE genotypes. (**A)** shows brain region of interest used to isolate cerebrovessel fractions. (**B)** shows identified total number of quantified spectra, peptide spectrum matches and non-redundant master protein groups from all TMT experiments. (**C)** Data shows level of expression for gene IDs associated with specific cell types identified in our proteomic analyses of the isolated cerebrovasculature (i.e. astrocytes, microglia, pericytes, endothelial cells, and smooth muscle cells). Data represent ratio expressed in arbitrary units. *Venn diagram* shows unique and overlapping significantly regulated proteins in the comparisons between (**D**) healthy controls from APOE2/2 vs APOE3/3, APOE3/4 vs APOE3/3, and APOE4/4 vs APOE3/3 genotypes, (**E)** Healthy matched Alzheimer’s disease cases vs controls from APOE2/3, APOE3/3, APOE3/4, and APOE4/4 genotypes. Asterisk in Venn diagram [*] denote unique non-overlapping proteins from each comparisons. **F**, **G and H** shows the violin plot for Amyloid plaque score, Tangle score and CAA score, respectively

Enriched cerebrovessels were isolated from the inferior frontal gyrus based on a previous protocol described by our group [59, 60]. Briefly, frozen 500 mg blocks of brain tissue from the inferior frontal gyrus was homogenized in ice-cold Hanks Buffered salt solution (HBBS) using a glass dounce homogenizer, and 6–8 passes of a Teflon pestle tissue grinder. A solution containing 40% dextran was added to the brain homogenate at an equal volume, to generate a final concentration of 20% dextran, which was subsequently centrifuged at 6000 g for 15 min at 4 °C. Three visible layers were produced after centrifugation; the top ‘paraenchyma fraction’ layer consisted of a compact mass, the bottom ‘cerebrovessel fraction’ layer consisted of a tissue pellet (Fig. 1A), and this was separated by a middle layer of translucent dextran interface consisting of the non-cell associated soluble fractions. For subsequent analyses we used the bottom layer consisting of the whole cerebrovascular fraction, containing vessels with a variety of sizes (microvessels, arterioles, etc.). This fraction was highly enriched in endothelial cells, pericytes, smooth muscle cells, and to a lesser extent astrocytes.

### Subcellular protein extraction from vascular homogenates

To each cerebrovascular pellet, we added 250 ul of ice cold phosphate buffered saline (PBS), followed by homogenization using a probe sonicator and subsequent centrifugation at 20,000 g for 5 min at 4 °C. Supernatants were collected in tubes to obtain the PBS-fraction. Pelleted samples were re-suspended in 250 ul of ice cold PBS containing 1 M sodium chloride, further sonicated and centrifuged at 20,000 g for 5 min at 4 °C. Supernatant was collected in a different tube and labeled as PBS-high salt fraction. The precipitant was resuspended in 250 ul of ice cold 20 mM Triethylamonium bicarbonate (TEAB) and 2% lithium dodecylsulphate anionic detergent, sonicated, and underwent centrifugation at 20,000 g for 5 min at 4 °C. Final supernatant was transferred to a new Eppendorf tube and labeled as the membrane protein pellet fraction. Twelve and a half microliters of 21× proteinase inhibitor cocktail was added to 250 ul of all the three fractions. To enhance the proteomic mining process, we used all three fractions—PBS, PBS-high salt and membrane fractions (i.e. cytosolic, nuclear and membrane proteins)—for the entire study.

### Trypsin digestion

BCA analyses was used to determine protein concentration prior to trypsin digestion. For the *PBS fraction,* 30 ug protein was added to 3× volume of acetone, and left to incubate at − 20 °C for 1 h. Following centrifugation at 14,000*g* for 1.5 min at room temperature, pelleted samples were brought up in 20 ul modified reduction alkylation buffer (MRAB) consisting of 20 mM TEAB at pH 8, 1% w/v sodium deoxycholate (SDC), 1 mM tris (2-carboxyethyl) phosphine (TCEP), and 2.5 mM 2-chloroacetamide (CAM). For the *PBS-high salt fraction,* 30ug protein was added to 1 in 5 parts of 20% w/v Trichloroacetic acid (TCA) and 3× volume of acetone, and left to incubate on ice for 1 h. Following centrifugation at 14,000*g* for 1.5 min at room temperature, pelleted samples were washed with 200 ul of acetone and pelleted material brought up in 20 ul MRAB. For the *membrane protein pellet fraction*, 30 ug protein was added to 20% of 100% w/v TCA, and left to incubate on ice for 1 h. Following centrifugation at 14,000*g* for 1.5 min at room temperature, pelleted samples were also washed with 200 ul of acetone and pelleted material resolubilized in 20 ul of MRAB. Validation of protein separation in all three protein fractions was conducted using sypro-red and Coomassie staining for total protein after polyacrylamide gel electrophoresis. Seven and a half microliters of all three protein fractions underwent trypsin digestion at a 1:100 enzymatic concentration. Firstly re-suspended samples in MRAB were incubated at 37 °C for 30 min; 7.5 ul of prepared activated trypsin solution (Promega, WI, USA) was added to re-suspended samples, and further incubated overnight at 37 °C while shaking mildly. Digested samples were stored at −80 °C prior to TMT labeling.

### Tandem mass tag (TMT) labeling strategy

We used a multiplexed isobaric labeling approach to allow for simultaneous identification and quantification of proteins from multiple biological samples. A 10-plex TMT labeling kit (ThermoScientific, NJ, USA) was used for analyses of protein samples from controls and AD with different APOE genotypes, with the control E3/E3 used as a reference sample per plex for normalization of data and as a reference point for the different runs. This labeling strategy allowed for all different groups (i.e. disease and genotype) to be randomized and analyzed within the same batch pool. All samples and isobaric label tags were handled blind to the experimenter. Twenty microliter aliquots of each label (dissolved in 20 ul of acetonitrile solution) were dried down in the speed vacuum and re-suspended in 25 mM TEAB made up in acetonitrile solution. Re-suspended labels were subsequently added to 10 ul of dried digested protein samples, and allowed to incubate for 1 h at room temperature, after which 1 ul of formic acid solution was added to stop the reaction. Labeled samples were pooled together in entire batches and subsequently dried in the speed vacuum. All three subcellular fractions were processed and this consisted of 18 batches of different 10-plex TMT pools.

### Sodium deoxycholate (SDC) and tetraethylammonium bromide (TEAB) clean up

To remove traces of SDC and TEAB, protein samples were re-suspended in 100 ul of 1% formic acid solution and centrifuged at 15,000 rpm for 1 min to allow separation into different phases. Supernatants were collected in new Eppendorf tubes, and 200ul of ethyl acetate was added, and centrifuged at 15,000 rpm with the upper organic layer discarded. This process was repeated three separate times, with the final lower phase taken to dryness in the speed vacuum. The resultant dried samples were re-solubilized in 100 ul of 0.1% formic acid.

### Purification and concentration of peptides

Prior to ultra-high pressure liquid chromatography (UHPLC), single step desalting, concentration and purification of peptides were conducted using 0.6 ul C18 resin Ziptips (ThermoScientific, NJ, USA). Briefly, ziptips pipette tips were used to remove contaminants by aspirating and dispensing in a solution of 0.1% formic acid made up in 50% acetonitrile (i.e. wetting buffer), and afterwards in a solution containing 0.1% formic acid (i.e. binding buffer). Ziptips were used for sample binding, by aspirating and dispensing through the samples multiple times. The resultant concentrated and purified labeled samples were aspirated in a solution of 5% methanol and 0.1% formic acid (i.e. washing buffer), followed by elution in a solvent containing 10 ul of 0.1% formic acid made up in 50% acetonitrile (wetting buffer). After desalting and concentrating peptides, final samples were dried and re-suspended in 20 ul of 0.1% formic acid and subsequently transferred into an auto-sampler vial, and analyzed by nano-Ultra-Performance Liquid Chromatography (UPLC) MS on a Q-Exactive Orbitrap instrument (ThermoScientific, NJ, USA).

### Chromatography and mass spectrometry (LC–MS/MS) methods

Pooled TMT-labeled peptides were analyzed using LC–MS/MS (Q-Exactive). A Thermo Easy UPLC was operated in a vented trap/elute configuration to separate TMT-labeled peptides. 5 µl of each re-constituted sample was loaded onto a 0.075 × 20 mm Pepmap C18 trapping column at 98% mobile phase A (MPA, 0.1% formic acid, water), and diverted to a 0.075 × 500 mm C18 Pepmap reversed phase column (2.0 µm particle) following an isocratic loading and washing step. Peptides were separated over a 4.5 h linear gradient of increasing mobile phase B (MPB, 0.1% formic acid, 99.9% acetonitrile) from 2 to 30 percent at 250 nl/min and 50 ℃. Xcalibur (Thermo) software was used to control the instrument in data dependent acquisition (DDA) mode. DDA settings for these MS experiments followed our previous work [57, 61], and were as follows: full-scan MS resolution = 140 000 full width at half maximum at 200 m/z, full-scan range = 380–1250 m/z, isolation width = 1.2 m/z, higher energy C-trap dissociation relative collision energy = 29, a minimum m/z setting of 100 m/z was used for all MS^2^ spectra, MS^2^ resolution = 35,000, dynamic exclusion = 180 s, and a Top 15 high/low duty cycle was used for precursor ion selection. Sample limitations did not allow for extensive two-dimensional (2D) fractionation of the labeled peptides, therefore we the use of a narrow isolation window and an ultra-long shotgun gradient was used to minimize the deleterious effects on quantitative accuracy that typically result from co-isolation of isobaric precursors.

### Data processing and statistical analysis of proteomics data

We surveyed our amalgamated data-files, and added other modifications to our search criteria if deemed necessary, using the PMi preview software. Preview results were used to choose the precursor and fragment ion mass tolerances (4-ppm, 0.02-Da, respectively) and dynamic modifications. We used the following settings to search the data using SEQUEST and BYONIC as the search algorithms, and Uniprot human database (FEB/2018). Dynamic modifications—Oxidation/ +15.995 Da (M), Methyl/ +14.016 Da (E), Deamidated/ +0.984 Da (N, Q), static modifications of TMT 10-plex/ +229.163 Da (N-Terminus, K), Carbamidomethyl + 57.021 (C). A co-isolation filter within PD 2.1 was set to 50% to exclude those PSMs which arose from MS/MS spectra that arose from co-isolation of precursor ions as a means to minimize the effects of signal distortion in the reporter ion region inherent to chimeric MS/MS. Only unique peptides were considered for our final quantification. We used the Percolator feature of Proteome Discoverer for SEQUEST, and used the target-decoy feature, to set a false discovery rate (FDR) of 0.01 for Byonic. The peptides passing this stringent cutoff FDR rate were subsequently exported for data cleaning and statistical analysis in JMP (SAS) software (version 15). Master proteins only underwent quantitative analysis if they were identified in at least 50% of the total number of plexes. JMP software employs a Shapiro–Wilk test for normality which was assessed prior to statistical analyses. Raw ion counts were *ln* transformed and analyzed by either two-way analyses of variance (ANOVA) (for AD and APOE interactions) or t-test (Control E2/E2 vs E3/E3, E3/E4 vs E3/E3, E4/E4 vs E3/E3  comparisons) to interrogate significantly regulated proteins between groups. Abundance ratio was generated by dividing each samples with the respective control E3/E3 sample used as a reference in the same plex for normalization of data (see Table 1). Log2 fold change and negative log 10 of the p value were subsequently uploaded into ingenuity pathway analyses (IPA) where molecules and pathways, diseases and biofunctions, associated networks and upstream regulators unique to each group comparison(s) were identified. Only master proteins with a significance level of 1.3 (i.e. negative log 10 of p value) were uploaded into IPA. We have deposited the mass spectrometry proteomic data into the ProteomeXchange Consortium via the PRIDE partner repository [62]. Our datasets can be located with the unique identifier—PXD023340.

### Ingenuity pathway analysis

All datasets of significantly modulated proteins from our group comparisons were uploaded into the Ingenuity Pathway Analysis software (IPA, Ingenuity® Systems [63]) to map them onto known networks of protein interactions in the knowledgebase. We further used the IPA knowledgebase to further determine the significantly regulated Canonical pathways/disease and biofunctions and biological significance of APOE and AD-dependent changes in the cerebrovasculature. Our core analysis settings involved the following—Ingenuity Knowledge base as reference set, maximum number of 35 molecules per network, and a maximum number of 25 networks for analysis. Only experimentally observed knowledge was considered in our analyses. We controlled for data sources, species, and tissue type/cell lines at the time of analysis in IPA. Core analysis identified canonical pathways shown to be significantly altered in response to APOE genotype and AD pathogenesis as a result of significantly regulated proteins represented in those pathways/biofunctions. Statistical significance of the relationship between uploaded dataset and the identified pathways/biofunctions was measured using two methods: (1) Ratio of the number of molecules from the data set that map to a pathway/biofunction divided by the total number of molecules in that pathway/ biofunction knowledgebase in IPA. (2) Fisher’s exact test, to calculate a p-value determining the probability that the association between the proteins in the dataset and the pathway/biofunctions are explained by chance alone. P values were considered to be significant in these studies when P < 0.01. Upstream regulator analysis was used to predict the upstream transcriptional master regulators in our proteomic dataset, and this was generated using the Ingenuity® Knowledge Base. An overlap P value was generated based on analyses of the significant overlap between proteins/genes in our dataset and known targets modulated by the transcriptional regulator or Upstream master regulator. The activation z-score algorithm was used to make predictions.

## Results

### Demographics and clinical background of patient population

In this study, we used 54 total brain cerebrovascular specimens from the inferior frontal gyrus of healthy controls (29) and Alzheimer's disease (AD) patients (26 cases)—(see Table 1). Both control and AD cases consisted of five different APOE genotypes, APOE2/2, APOE2/3, APOE3/3, APOE3/4, and APOE4/4. APOE2/2 cases were low in sample size for the AD group, and as such were removed from our analyses. On average control patients (72.9 ± 3.49) were younger than AD cases (85.24 ± 2.10). This was driven by the younger age of APOE4/4 controls (45.83 ± 4.39). Most of the other cases were comprised of septuagenarians and octogenarians. Each group consisted of mixed genders, and on average, there were more females in both controls (62.1%) and AD (61.5%) cohorts.

Alzheimer's disease cases were classified based on the Braak staging, amyloid plaque score, and Tangle score. The majority of AD cases consisted of Braak stage IV–VI, Tangle score ranging from 8 to 15, and plaque score between 7 and 14 (Table 2). E4/E4 cases had the worst Braak staging, and plaque score (Table 2; see also Fig. 1F and 1G). E4/E4 also had the highest mean CAA score (8), while E3/E3 had the lowest score (Table 2; see also Fig. 1H). E3/E3 and E4/E4 AD groups had the worst mean final MMSE scores (6 and 11 respectively), and the E2/E3 and E3/E4 (both approx.18) had the highest mean final MMSE scores (Table 2). E4/E4 AD patients also had the smallest brain weight (959 ± 18.1) of all cases (Table 2).

### Proteomic profiles, cell type changes and altered canonical pathways in enriched cerebrovascular tissue from the inferior frontal gyrus of control patients from  different APOE genotypes

A 10-plex TMT isobaric tag approach was used to study the proteomic profiles of brain cerebrovascular tissue from the inferior frontal gyrus of AD cases and controls. We identified a total of 13,760 total peptide spectrum matches and 2036 non-redundant master protein groups from all TMT experiments (Fig. 1B). To determine the cell type constituents of our enriched cerebrovascular tissue we identified cell specific protein markers in our proteomic dataset using the single cell sequencing resource from the PanglaoDB omic database. We measured the relative protein expression levels of these specific markers associated with these different cell types in control samples, and observed that there was a relatively high expression and abundance levels of markers associated with pericytes (AOC3, COL1A1, MYO1B) and endothelial cells (CLEC14A, VWF, Cav1, PLEC) in our enriched cerebrovascular fractions (Fig. 1C). This was followed by smooth muscle cells (EHD2, ITGA1, MYL9) and astrocytes (GFAP, AQP4, S100β, APOE). Neurons and microglia related proteins were least expressed in our cerebrovascular fractions.

**Fig. 2:**
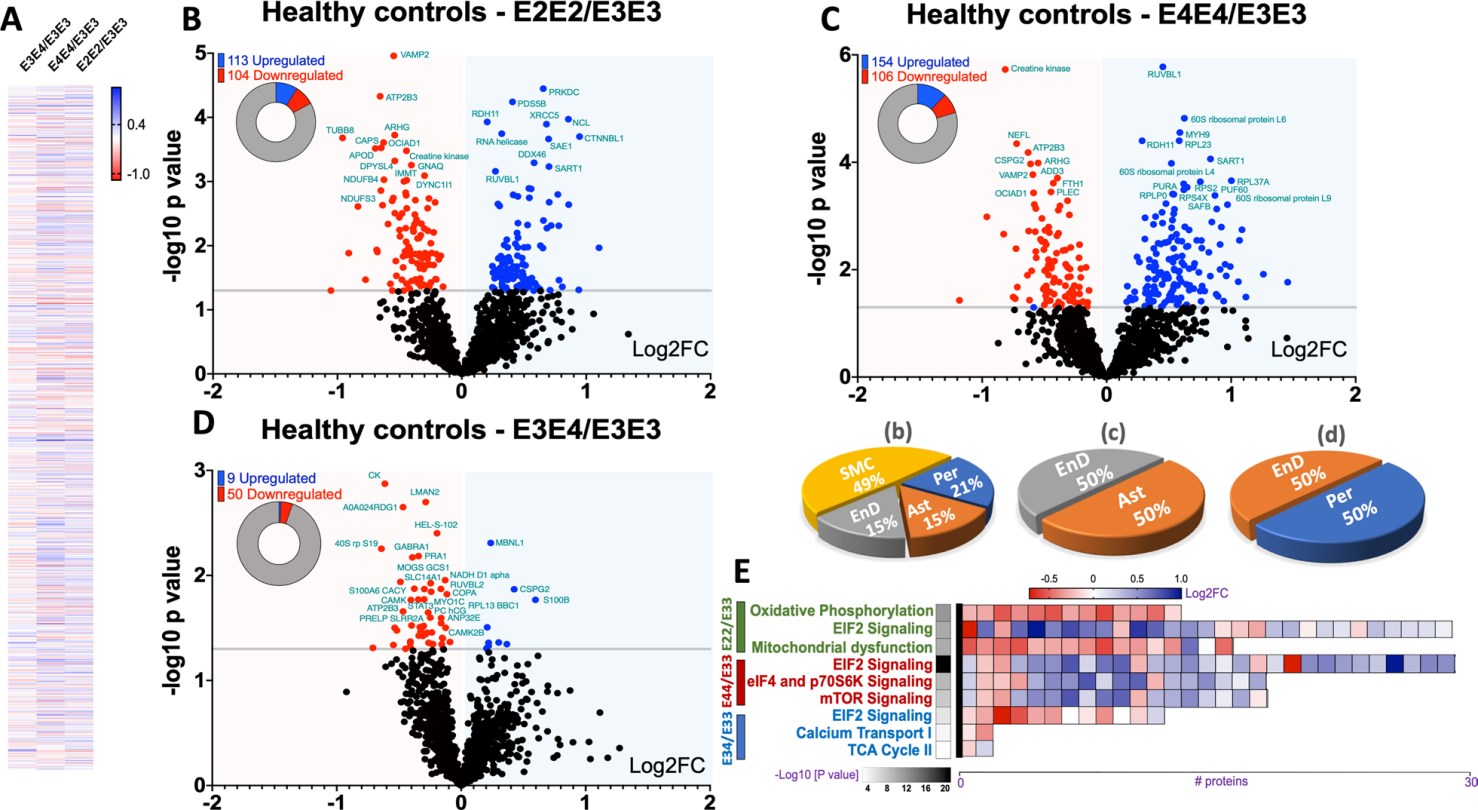
Differentially expressed proteins and cell type origin in healthy controls with  different APOE genotypes. Heat map (Log2FC) of all master proteins identified across the three different genotypes of interests compared to APOE3/E3 controls (**A**). Volcano plot of differentially expressed proteins in healthy controls from APOE2/E2 vs APOE3/E3 genotypes (**B**), APOE4/E4 vs APOE3/E3 genotypes (**C**), and APOE3/E4 vs APOE3/E3 genotypes (**D**). Pie chart inset of graphs shows up/down-regulated proteins from each comparisons. Pie Chart shows origin of cell types where significant (cerebrovascular cell specific) proteins are observed  between APOE2/E2 vs APOE3/E3 controls (**b**), APOE4/E4 vs APOE3/E3 controls (**c**), and APOE3/E4 vs APOE3/E3 controls (**d**). Values are generated from the ratio of significantly altered cerebrovascular cell specific proteins identified within our entire control datasets, and further expressed as a percentage of all 4 cerebrovascular cell types. *EnD—endothelial cells, Ast—astrocytes, Per—Pericytes, SMC—smooth muscle cells*. (**E)** shows heat map of the top 3 altered pathways  from the three different APOE genotype comparisons and the corresponding number of significant proteins and their Log2 fold change expression level

Prior to comparing the disease and APOE genotype interaction effects in our proteomic analyses, we first interrogated any underlying APOE genotype effects in the healthy controls that may drive disease outcome in AD patients. We focused this analyses on healthy control cohorts from E2/E2, E3/E3, E3/E4 and E4/E4 groups. A T-test approach was used to analyze the master proteins to identify significant changes in unique and common proteins altered between APOE4/4 vs APOE3/3, APOE3/E4 vs APOE3/E3, and APOE2/2 vs APOE3/3 genotypes. Statistical analyses identified a total of 217 master proteins significantly changing in APOE2/2 vs APOE3/3, 260 in APOE4/4 vs APOE3/3, and 59 in APOE3/4 vs APOE3/3 (Fig. 1D**—**for a full list of significantly regulated proteins, see Additional files 1, 2, 3: Tables S1–S3). Heat map of all proteins identified, and the distribution of Log2 fold change in correlation with negative log10 p value can be found in Fig. 2A–D.

We interrogated the origin of cell types showing the most significant changes in cerebrovascular cell specific markers within our control datasets, and expressed this as a percentage of all 4 cerebrovascular cell types. We observed that most of the significant changes in our proteomic datasets between APOE2/2 vs APOE3/3 controls were associated with endothelial cell (15%), pericyte (21%), astrocyte (15%), smooth muscle cell specific proteins (49%) **(**Fig. 2b). We also revealed that most of the significant changes in our proteomic datasets between APOE4/4 vs APOE3/3 controls were associated with endothelial cell (50%) and astrocyte specific proteins (50%) (Fig. 2c). While significant changes in cell specific markers between APOE3/4 vs APOE3/3 controls  were mainly associated with endothelial cell (50%) and pericyte specific proteins (50%) (Fig. 2d).

**Fig. 3:**
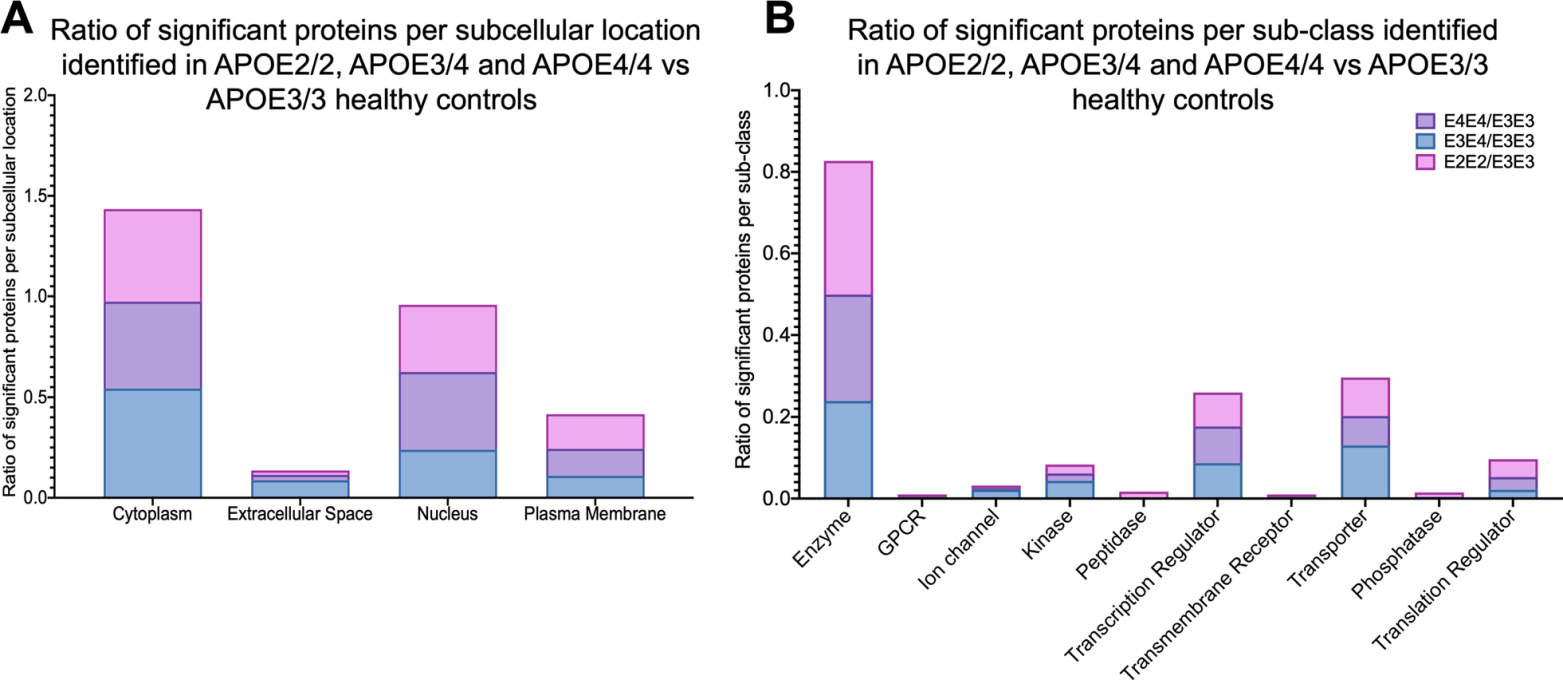
Subcellular localization and sub-class of proteins identified in the cerebrovessels of the inferior frontal gyrus in APOE2/2, APOE3/4 and APOE4/4 vs APOE3/E3  healthy controls. **A** shows ratio of significant proteins localized to the cytoplasm, extracellular space, nucleus and plasma membranes.** B** shows ratio of significant proteins and their corresponding sub-classification

We categorized the significant proteins identified above into their subcellular origin, and functional sub-groups (i.e. enzymes, receptors, transporters etc.). We noted a fairly similar change across the three different genotype group comparisons (see Fig. 3). Significantly regulated proteins were typically of cytoplasmic and nuclear origins (Fig. 3A), and were primarily enzymes, transcription regulators and transport/carrier proteins (Fig. 3B).

**Fig. 4:**
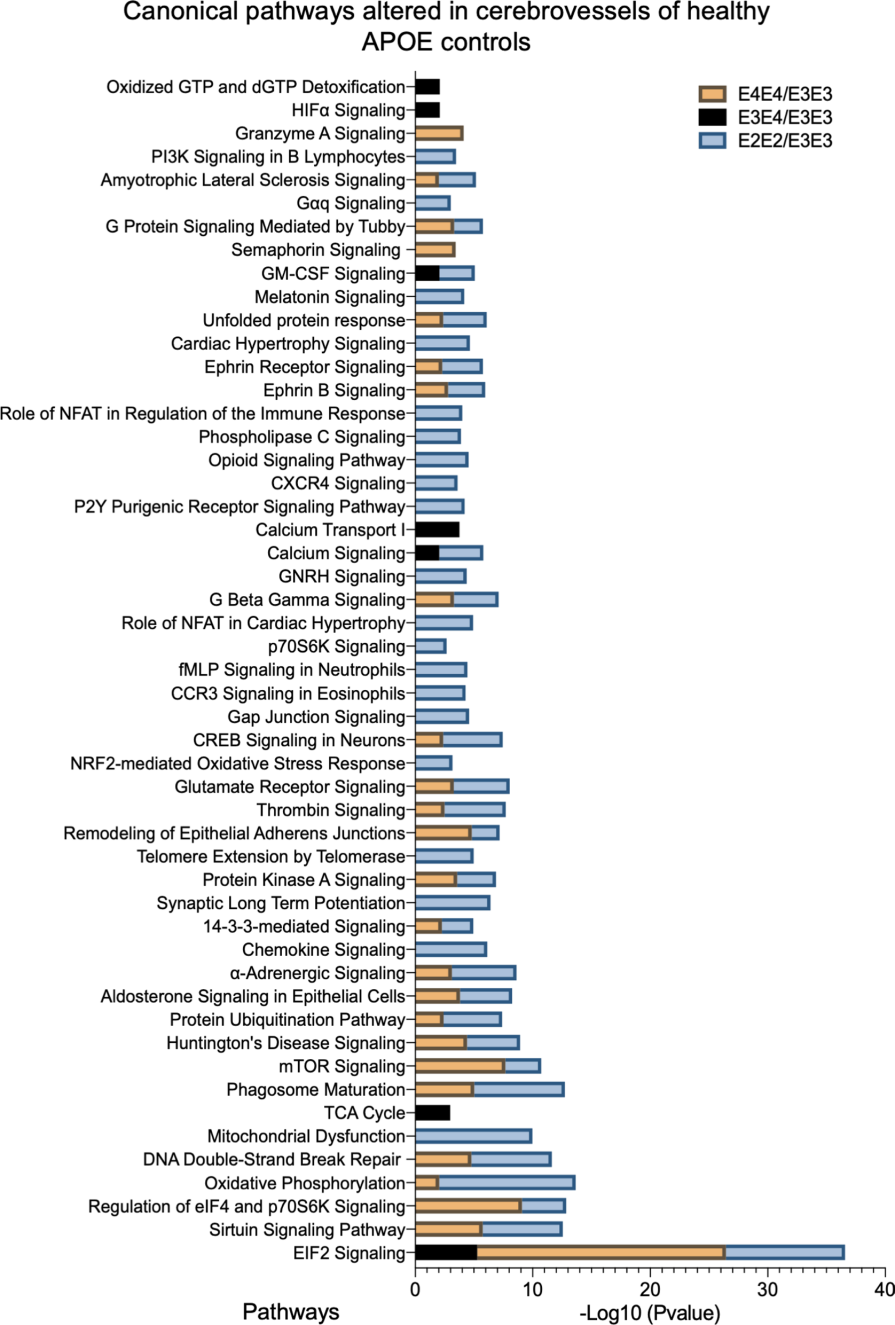
Canonical pathways modulated in the cerebrovasculature of the inferior frontal gyrus in healthy controls with different  APOE genotypes cases. Identified canonical pathways were generated from the list of significantly modulated proteins between healthy controls from APOE2/E2 vs APOE3/E3, APOE3/E4 vs APOE3/E3, and APOE4/E4 vs APOE3/E3 genotypes using Ingenuity pathway analyses. Values represent negative log 10 of FDR adjusted P value after Fischer’s test and Benjamin Hochberg correction. Significant cut-off is set at $\ge$ 2. APOE2/E2 vs APOE3/E3 (45 pathways identified), APOE4/E4 vs APOE3/E3 (25 pathways identified), and APOE3/E4 vs APOE3/E3 (7 pathways identified)

Ingenuity pathway analyses revealed numerous pathways significantly impacted within control groups from different  APOE genotype (Fig. 4). The top 3 pathways altered in APOE4/4 vs APOE3/3 controls include downregulation of EIF2 signaling, regulation of EIF4 and p70S6k signaling, and mTOR signaling. Top 3 altered pathways in APOE2/2 vs APOE3/3 controls were oxidative phosphorylation, EIF2 signaling and mitochondrial dysfunction. While in APOE3/4 vs APOE3/3 control, downregulation of EIF2 signaling, altered Calcium transport and TCA cycle were the top 3 pathways signficantly altered. Heat map in Fig. 2E, shows the number of proteins associated with the top 3 pathways, and their direction of change.

Of the significantly regulated proteins identified from our APOE genotype comparisons, we identified 85 out of 217 to be unique to the APOE2/2 vs APOE3/3 group alone, 128 out of 260 to the APOE4/4 vs APOE3/3 group, and 40 out of 59 proteins to the APOE3/4 vs APOE3/3 group (see Venn diagram—Fig. 1D). We used the ingenuity pathway analyses to identify the pathways unique to only APOE2/2 vs APOE3/3, these included mitochondrial dysfunction, chemokine signaling, fMLP signaling in immune cells, Phospholipase C, GNRH and CCR3 signaling (Fig. 4). We identified TCA cycle, Calcium transport I, HIF $\alpha$ signaling and oxidized GTP and dGTP detoxification as pathways unique to only the APOE3/4 vs APOE3/3 genotype comparison (Fig. 4). While semaphorin A signaling and granzyme A signaling were the only pathways unique to the APOE4/4 vs APOE3/3 comparison (Fig. 4).

**Fig. 5:**
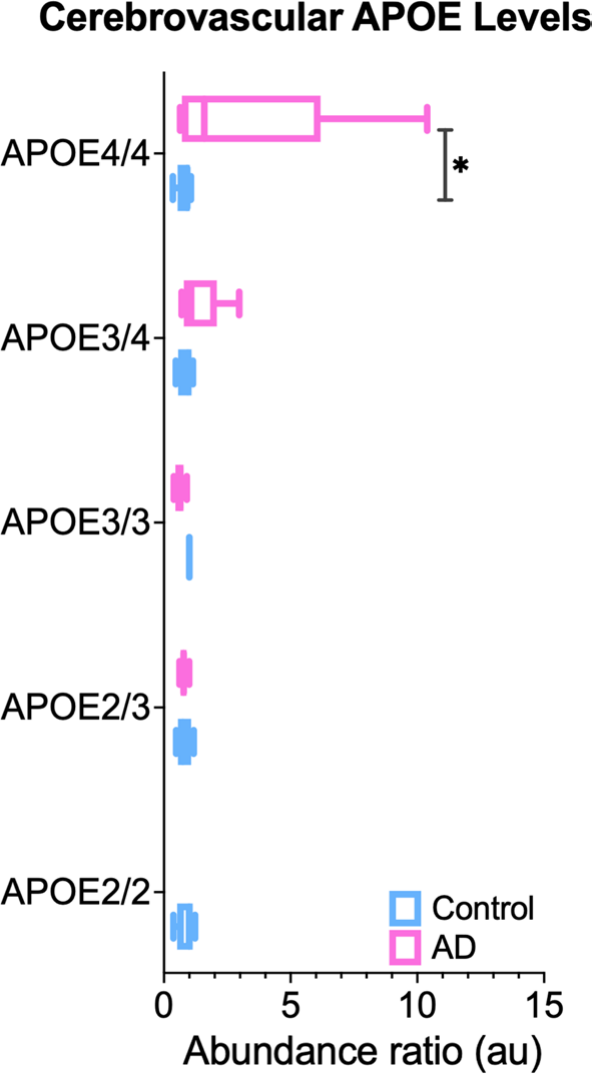
APOE levels in the cerebrovasculature of the inferior frontal gyrus in AD cases vs healthy controls from different APOE genotypes. Asterisk denotes *P < 0.05

Our interrogation of APOE levels in the cerebrovascular fractions revealed no significant changes across the genotypes in healthy non-demented cases (Fig. 5).

### Proteomic profiles, cell type changes  and canonical pathways altered in cerebrovascular tissue from the inferior frontal gyrus of control vs AD cases from different APOE genotypes

**Fig. 6:**
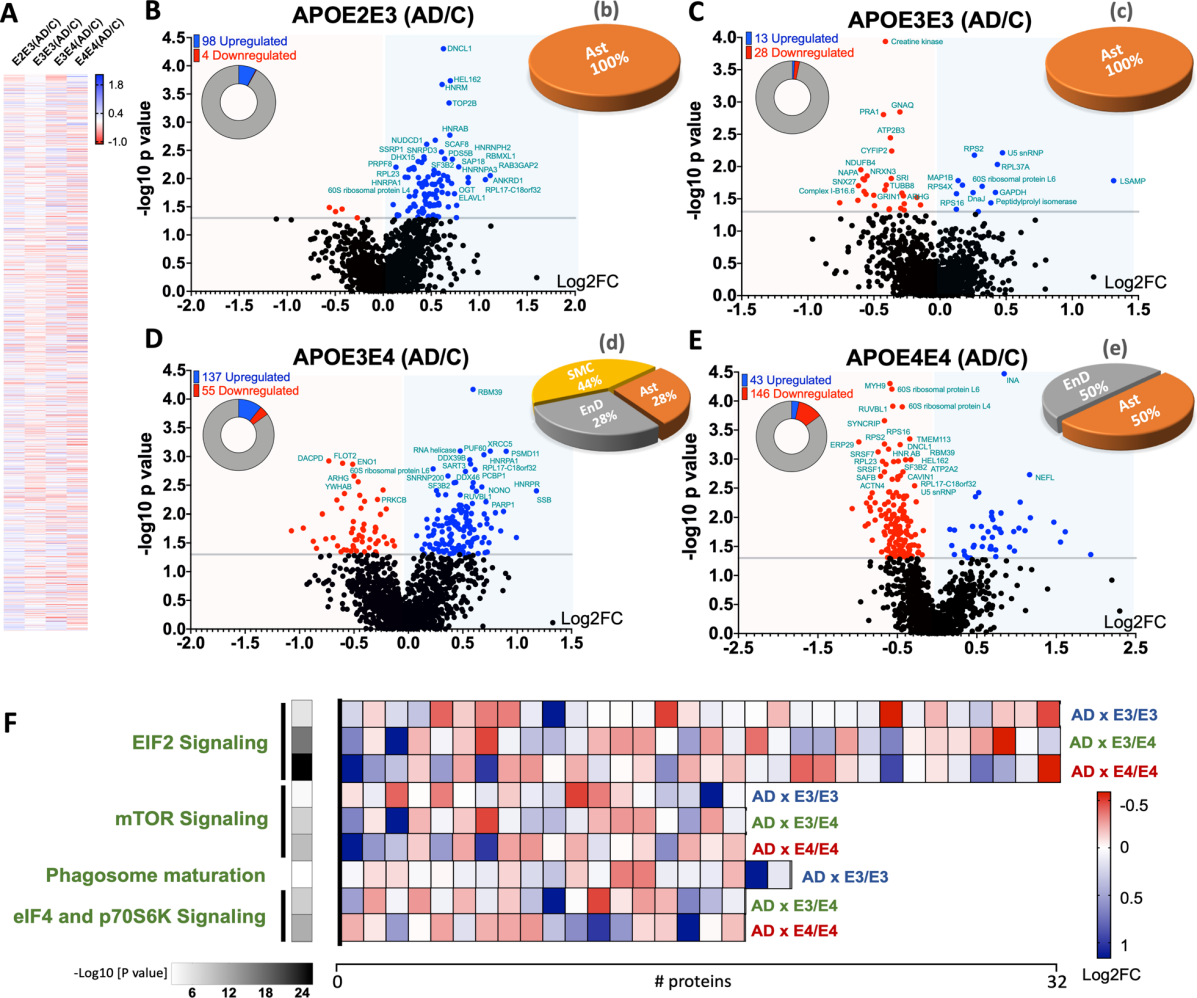
Differentially expressed proteins and cell type origin in the cerebrovasculature of the inferior frontal gyrus between Alzheimer's disease (AD) patients and controls from different APOE genotype backgrounds. Heat map (Log2FC) of all master proteins identified between AD vs controls cases across the four different APOE genotypes (**A**). Volcano plot of differentially expressed proteins in AD vs matched controls from APOE2/E3 (**B**), APOE3/E3 (**C**), APOE3/E4 (**D**), and APOE4/E4 (**E**) genotypes. Pie chart inset of graphs shows up/down-regulated proteins from each comparisons*.* Pie Chart shows origin of cell types where significant (cerebrovascular cell specific) proteins are observed from the comparisons between AD vs control cases from APOE2/E3 (**b**), APOE3/E3 (**c**), APOE3/E4 (**d**), and APOE4E/4 (**e**) genotypes. Values are generated from the ratio of significantly altered cerebrovascular cell specific proteins identified within our AD vs control datasets, and expressed as a percentage of all 4 cerebrovascular cell types. . *EnD—endothelial cells, Ast—astrocytes, Per—Pericytes, SMC—smooth muscle cells.* (**F)** shows heat map of the top 3 pathways for the four different genotype comparisons and the corresponding number of significant proteins and their Log2 fold change expression level

A two way ANOVA approach was used to identify significant changes in master proteins showing a genotype and disease interaction. Statistical analyses identified a total of 102 master proteins in the E2/E3 AD cases vs controls, 41 proteins in the E3/E3 AD cases vs controls, 192 proteins in the E3/E4 cases vs controls, and 189 proteins in the E4/E4 cases vs controls   (Fig. 1E—for a full list of significantly regulated proteins see Additional files 4, 5, 6, 7: Tables S4–S7). A list of the Top 25 proteins signficantly altered in E3/E4 and E4/E4 AD cases vs controls are provided (see Tables 4 and 5). Heat map of all proteins identified, and the related volcano plots  can be found in Fig. 6A–D.

We interrogated the origin of cell types showing the most significant changes in cerebrovascular cell specific markers within our AD vs control datasets  to demonstrate the impact of our proteomic changes at the single cell level (Fig. 6b–e). We observed that the origin of cell specific protein markers demonstrating significant changes in E2/E3 AD cases vs controls were derived primarily from astrocytes (100%) (Fig. 6b). We observed a similar outcome in E3/E3 cases vs controls  (Fig. 6c). In the E3/E4 cases, we observed changes in—smooth muscle cell derived proteins (44%), astrocytes and endothelial cell specific proteins (each 28%) (Fig. 6d). While in the E4/E4 cases, we observed changes implicating 2 cell types, with 50% attributed to astrocytes and 50% to endothelial cells (Fig. 6e).

**Fig. 7:**
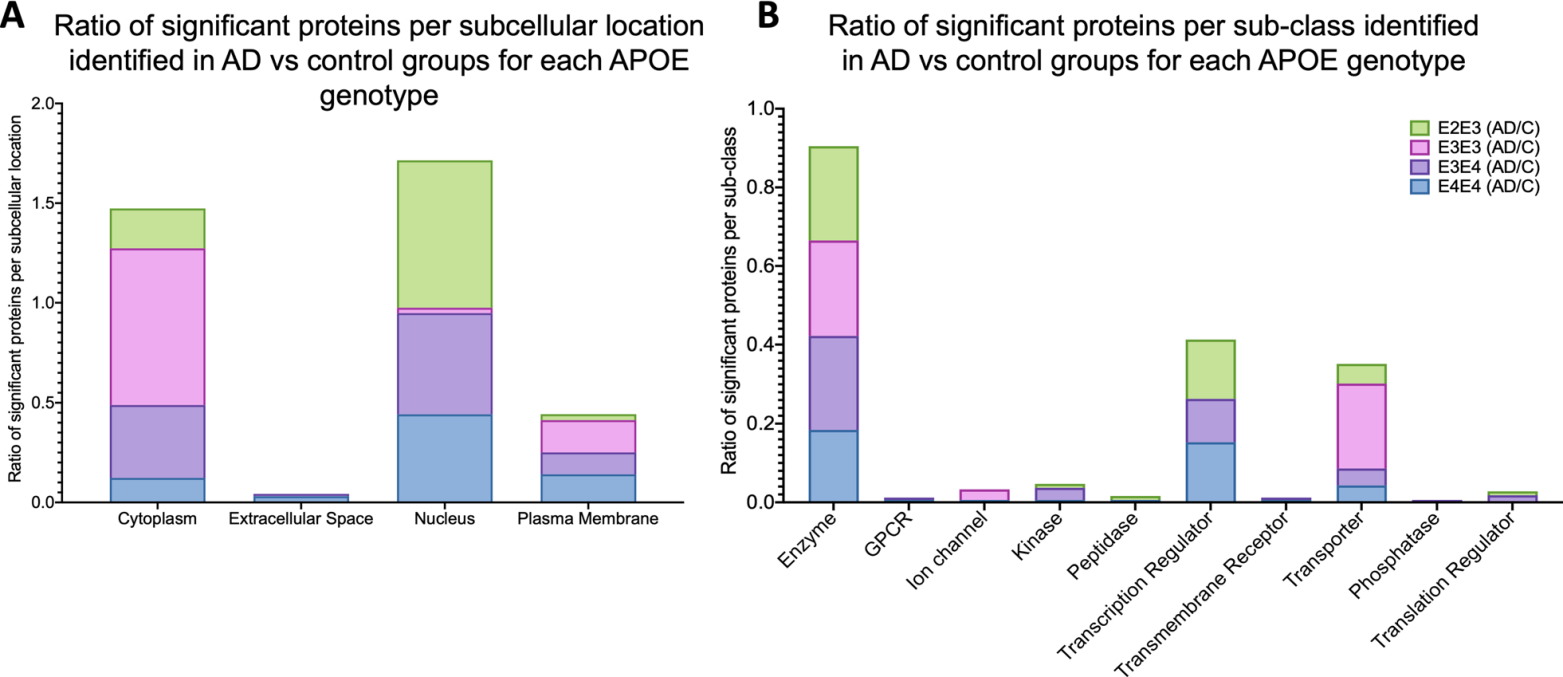
Subcellular localization and sub-class of proteins identified in the cerebrovessels of the inferior frontal gyrus in AD cases vs controls from APOE2/E3, APOE3/E3, APOE3/E4 and APOE4/E4 genotypes. (**A)** shows ratio of significant proteins localized to the cytoplasm, extracellular space, nucleus and plasma membranes. (**B)** shows ratio of significant proteins and their corresponding sub-classification

We stratified the significant proteins identified from the disease x genotype interactions into their subcellular origins and functional sub-groups. Significantly regulated proteins identified between AD vs controls from E2/3 and E4/4 genotypes were primarily localized to the nucleus, while E3/3 cases were localized to the cytoplasm (Fig. 7A). With respect to the functional sub-groups, significantly regulated proteins identified between AD vs control cases  from E2/3, E3/4 and E4/4 genotypes were primarily enzyme-related proteins and transcriptional regulator proteins, while E3/3 cases consisted of mainly enzyme-related and transport/carrier proteins (Fig. 7B).

Ingenuity pathway analyses of significantly regulated proteins revealed numerous pathways impacted by disease and APOE genotype interactions (Table 3). The Top 3 pathways identified  between AD vs controls from E3/E4 and E4/E4 genotypes include EIF2 signaling, regulation of EIF4 and p70S6k signaling and mTOR signaling. In the E3/E3 AD vs control groups, we identified EIF2 signaling, mTOR signaling and Glutamine Biosynthesis I as the top 3 pathways. While from the E2/E2 AD vs control groups the Top 3 pathways identified were spliceosomal cycle, DNA methylation and transcriptional repression signaling and Base excision repair (BER) pathways involved in the repair of damaged DNA during the cell cycle. Heat map in Fig. 6F, shows the number of proteins associated with the top 3 significantly modulated pathways from the four different disease × APOE genotype interactions described above, and their direction of change.

**Table 3:**
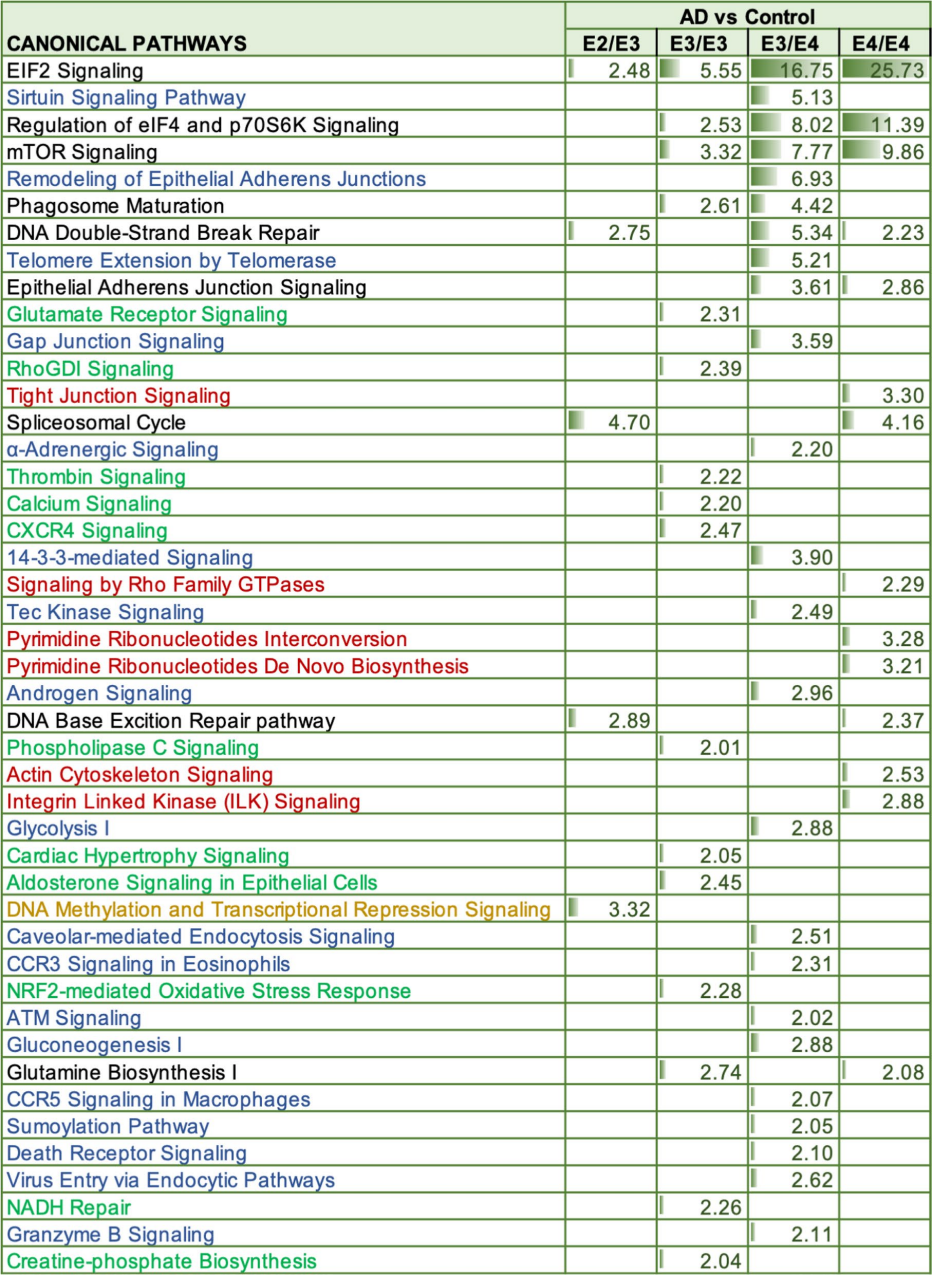
Canonical pathways modulated in the cerebrovasculature of the inferior frontal gyrus between Alzheimer's disease (AD) patients and controls from different APOE genotype backgrounds

Identified canonical pathways were generated from the list of significantly modulated proteins between AD vs controls from APOE2/E3, APOE3/E3, APOE3/E4 and APOE4/E4 genotypes using Ingenuity pathway analyses. Values represent negative log 10 of FDR adjusted P value after Fischer’s test and Benjamin Hochberg correction. Significant cut-off is set at $\ge$ 2. AD vs control APOE2/E3 genotype (5 pathways identified), AD vs control APOE3/E3 genotype (16 pathways identified), AD vs control APOE3/E4 genotype (24 pathways identified), and AD vs control APOE4/E4 genotype (14 pathways identified). *Orange highlights—unique to E2/E3 only, Green highlights—unique to E3/E3 only, Blue highlights—unique to E3/E4 only, Red highlights—unique to E4/E4 only*

Of the significantly regulated proteins showing a disease and APOE genotype interaction, we identified only 8 out of 102 to be unique to the APOE2/3 group alone, 9 out of 41 to the APOE3/3 group, 33 out of 192 to the APOE3/4 group, and 66 out of 189 proteins to the APOE4/4 group (see Venn diagram—Fig. 1E). Ingenuity pathway analyses generated pathways unique to disease and (E4/E4) genotype interaction alone, some of which included Tight junction signaling, neucleobases (pyramidine) synthesis, actin cytoskeleton signaling and Integrin linked kinase signaling, and from the E3/E4 group the unique pathways identified were sirtuin signaling pathway, remodeling of adheren junctions, Telemore extension, 14-3-3 mediated signaling, Caveolae mediated endocytosis signaling and CCR3 signaling (Table 3). CXCR4 signaling, NRF-2 mediated oxidative stress response, Thrombin, and aldosterone signaling were some of the top pathways unique to the E3/E3 group  (Table 3). Only DNA methylation and transcriptional repression signaling pathway were  unique to the disease and (E2/E3) genotype interaction (Table 3).

Our assessment of APOE levels in the cerebrovascular tissue showed a significant increase in AD vs controls from the APOE4/4 genotype, and a trend towards increase in APOE3/4 cases (Fig. 5).

**Fig. 8:**
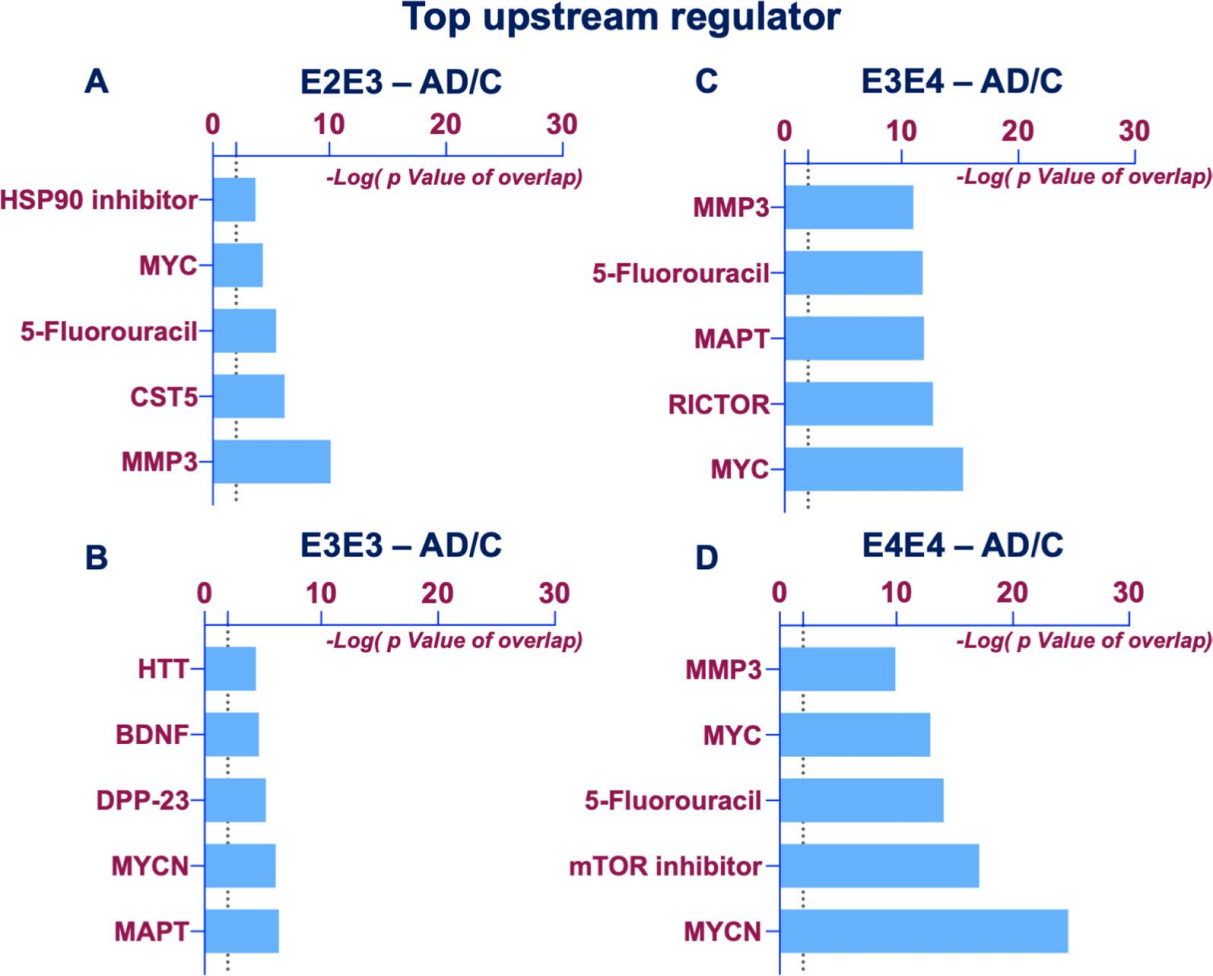
Upstream regulators implicated in the proteomic changes observed in the cerebrovasculature of the inferior frontal gyrus between Alzheimer's disease (AD) patients and controls from different APOE genotype backgrounds. Histograms shows Top 5 significantly altered upstream regulators identified by ingenuity pathway analyses between AD vs  controls from APOE2/E3 (**A**), APOE3/E3 (**B**), APOE3/E4 (**C**) and APOE4/E4 (**D**) genotypes. Data represents negative Log10 of the FDR adjusted “overlap” P value after Fischer’s test and Benjamin Hochberg correction. Overlap P values are generated based on the significant overlap between dataset proteins/genes and known targets regulated by a transcription factor/upstream regulator. Cut of level for significantly altered upstream regulators was set at $\ge$ 2 (i.e. −log 10[P value])

We interrogated the Top 5 Upstream Regulators mediating the changes observed between disease x APOE genotype interactions (Fig. 8). From the E2/E3 group, IPA identified a significant overlap in our dataset and known targets regulated by these five Upstream regulators, MMP3 (matrix metalloproteinase protein 3), CST5 (cystatin-D), 5-Fluorouracil (pyrimidine analogue blocking DNA synthesis), MYC (Proto-Oncogene, BHLH Transcription Factor), tanespimycin (HSP90 inhibitor) (Fig. 8A). From the E3/E3 group we revealed MAPT (microtubule associated protein tau), MYCN (N-myc Proto-Oncogene, BHLH Transcription Factor), DPP-23 (apoptosis activator IX), BDNF (brain derived neurotrophic factor), and HTT (huntingtin) as the top 5 upstream regulators  (Fig. 8B), while MYC, RICTOR (rapamycin-insensitive companion of mammalian target of rapamycin), MAPT, 5-fluorouracil, and MMP3 were identified in the E3/E4 group (Fig. 8C). MYCN, rapamycin (mTOR inhibitor), 5-fluorouracil, MYC, and MMP3 were also identified as top upstream regulators in the E4/E4 groups  (Fig. 8D) (Tables 4, 5).

**Table 4:** List of Top 25 proteins significantly regulated in the cerebrovasculature of the inferior frontal gyrus in Alzheimer's disease vs controls from APOE3/4 genotype

Gene ID	Label	Protein name	Biological function	log 2FC	P value
AINX	Q16352	Alpha-internexin (Alpha-Inx) (66 kDa neurofilament protein)	Structural constituent of cytoskeleton	0.845	3.38E−05
MYH9	P35579	Myosin-9 (Cellular myosin heavy chain, type A) (Myosin heavy chain 9)	Actin binding	− 0.593	5.01E−05
Q59GY2	Q9HBB3	60S ribosomal protein L6	Structural constituent of ribosome	− 0.569	6.24E−05
B2R8Z8	Q59GY2	Ribosomal protein L4 variant (Fragment)	RNA binding	− 0.558	1.23E−04
A0A024R333	B5BUB1	RuvB-like helicase (EC 3.6.4.12) (Fragment)	Chromatin binding	− 0.438	1.26E−04
ERP29	B2R8Z8	cDNA, FLJ94136, synaptotagmin binding, cytoplasmic RNA interacting protein (SYNCRIP)	Chaperone binding	− 0.663	2.19E−04
RS2	A0A024R333	Transmembrane protein 113, isoform CRA_a	Cadherin binding	− 0.344	4.49E−04
Q6FGH9	P30040	Endoplasmic reticulum resident protein 29 (ERp29) (Endoplasmic reticulum resident protein 28)	Dynein intermediate chain binding	− 0.985	5.07E−04
RS16	P15880	40S ribosomal protein S2 (40S ribosomal protein S4)	RNA binding	− 0.662	5.51E−04
SRSF7	Q6FGH9	Dynein light chain	Protein domain specific binding	− 0.465	5.64E−04
A0A0S2Z3L2	P62249	40S ribosomal protein S16 (Small ribosomal subunit protein uS9)	ATP binding	− 0.611	6.75E−04
Q53F64	Q16629	Serine/arginine-rich splicing factor 7 (Splicing factor 9G8) (Splicing factor, arginine/serine-rich 7)	RNA binding	− 0.742	7.52E−04
V9HWK4	A0A0S2Z3L2	ATPase Ca++ transporting cardiac muscle slow twitch 2 isoform 1	Double-stranded RNA binding	− 0.331	1.02E−03
A8KAP3	Q14498	RNA-binding protein 39 (CAPER alpha) (Hepatocellular carcinoma protein 1)	GTPase activity	− 0.396	1.03E−03
A8K329	A0A024R1Q8	Ribosomal protein L23, isoform CRA_b	RNA binding	− 0.488	1.09E−03
NFL	Q53F64	Heterogeneous nuclear ribonucleoprotein AB isoform a variant (Fragment)	Structural constituent of cytoskeleton	− 0.686	1.10E−03
CAVN1	V9HWK4	Epididymis luminal protein 162	Identical protein binding	− 0.547	1.11E-03
J3KTL2	Q13435	Splicing factor 3B subunit 2	Protein kinase B binding	− 0.644	1.23E−03
ACTN4	A8KAP3	cDNA FLJ78483, elongation factor Tu GTP binding domain containing 2 (EFTUD2)	Actin filament binding	− 0.429	1.66E−03
RL24	A8K329	cDNA FLJ76656, scaffold attachment factor B (SAFB)	Cadherin binding	− 0.662	1.67E−03
OGT1	P07196	Neurofilament light polypeptide (NF-L) (68 kDa neurofilament protein)	Phosphatidylinositol-3,4,5-trisphosphate binding	1.164	1.86E−03
B1PS43	Q6NZI2	Caveolae-associated protein 1 (Cavin-1)	Actin filament binding	− 0.463	1.94E−03
RL3	J3KTL2	Serine/arginine-rich-splicing factor 1	Structural constituent of ribosome	− 0.711	1.98E−03
B4DEG4	O43707	Alpha-actinin-4 (Non-muscle alpha-actinin 4)	RNA binding	− 0.495	2.02E−03
PLEC	A0A0A6YYL6	Protein RPL17-C18orf32	Structural constituent of cytoskeleton	− 0.565	2.23E−03

Tables show the biological functions of the top 25 significant proteins between AD vs control from the APOE3/E4 genotype

**Table 5:** List of Top 25 proteins significantly regulated in the cerebrovasculature of the inferior frontal gyrus in Alzheimer's disease vs controls from APOE4/4 genotypes

Gene ID	Label	Protein name	Biological function	log 2FC	P value
RBM39	Q14498	RNA-binding protein 39 (CAPER alpha) (Hepatocellular carcinoma protein 1)	RNA binding	0.594	0.000
Q59F66	Q59F66	DEAD box polypeptide 17 isoform p82 variant (Fragment)	Nucleic acid binding	0.476	0.001
XRCC5	P13010	X-ray repair cross-complementing protein 5	Damaged DNA binding	0.752	0.001
PSD11	O00231	26S proteasome non-ATPase regulatory subunit 11 (26S proteasome regulatory subunit RPN6)	Structural molecule activity	0.899	0.001
A0A024RAZ7	A0A024RAZ7	Heterogeneous nuclear ribonucleoprotein A1, isoform CRA_b	RNA binding	0.694	0.001
DX39B	Q13838	Spliceosome RNA helicase DDX39B	ATPase activity	0.564	0.001
E7EMK3	E7EMK3	Flotillin-2	Protease binding	− 0.731	0.001
B2R673	B2R673	Dihydrolipoamide acetyltransferase component of pyruvate dehydrogenase complex	Transferase activity, transferring acyl groups	− 0.605	0.001
SART3	Q15020	Squamous cell carcinoma antigen recognized by T-cells 3 (SART-3)	Histone binding	0.571	0.001
ENOA	P06733	Alpha-enolase	Cadherin binding	− 0.512	0.001
Q9HBB3	Q9HBB3	60S ribosomal protein L6	DNA binding	0.227	0.002
PUF60	Q9UHX1	Poly(U)-binding-splicing factor PUF60 (60 kDa poly(U)-binding-splicing factor)	Cadherin binding	0.612	0.002
Q53SS8	Q53SS8	Epididymis secretory protein Li 85 (Poly(RC) binding protein 1)	DNA-binding transcription factor activity	0.524	0.002
A0A0A6YYL6	A0A0A6YYL6	Protein RPL17-C18orf32	Structural constituent of ribosome	0.364	0.002
Q6ICQ8	Q6ICQ8	ARHG protein (Ras homolog gene family, member G (Rho G))	GTPase activity	− 0.500	0.002
B5BU24	B5BU24	14-3-3 protein beta/alpha		− 0.462	0.003
SF3B2	Q13435	Splicing factor 3B subunit 2	RNA binding	0.435	0.003
A0A0C4DG89	A0A0C4DG89	Probable ATP-dependent RNA helicase DDX46	ATP binding	0.595	0.003
U520	O75643	U5 small nuclear ribonucleoprotein 200 kDa helicase	Helicase activity and ATP binding	0.420	0.003
B2R7W4	B2R7W4	cDNA, FLJ93632, heterogeneous nuclear ribonucleoprotein R (HNRPR), mRNA	RNA binding	0.675	0.003
A0A0S2Z4Z9	A0A0S2Z4Z9	Non-POU domain containing octamer-binding isoform 1 (Fragment)	RNA binding	0.588	0.003
KPCB	P05771	Protein kinase C beta type (PKC-B) (PKC-beta)	Calcium channel regulator activity	− 0.233	0.004
B5BUB1	B5BUB1	RuvB-like helicase (EC 3.6.4.12) (Fragment)	5'-3' DNA helicase activity	0.258	0.004
B5BUB5	B5BUB5	Autoantigen La (Fragment)	RNA binding	1.177	0.004
PARP1	P09874	Poly [ADP-ribose] polymerase 1 (PARP-1)	DNA binding	0.625	0.004

Tables show the biological functions of the top 25 significant proteins between AD vs controls cases from the APOE4/E4 genotype background

## Discussion

Very few studies have explored the influence of APOE genotype on the pathobiological changes to the cerebrovasculature in the pathogenesis of AD. We hypothesized that APOE genotype specific molecular aberrations (at the protein level) in the cerebrovasculature, may compromise their physiological properties and essential functions, and thus explain the influence of APOE genotype on cerebrovascular dysfunction in AD pathogenesis.

To address this, we employed our state-of-the-art proteomic platform to conduct a detailed unbiased characterization of changes in protein expression levels, and molecular pathways significantly altered in cerebrovessels isolated from the inferior frontal gyrus of control and AD brains from five different APOE genotype backgrounds. The cerebrovessel fraction expressed very high levels of endothelia and pericyte cell specific markers, and a moderate expression level of astrocyte and smooth muscle cell specific markers. From our proteomic analyses we revealed unique changes in proteins and molecular pathways driven by APOE genotype that could explain the vulnerability of the cerebrovasculature in the pathological sequelae of AD.

### APOE specific effects in non-demented cases

The insidious cascades of events triggered in the brains of Alzheimer's disease patients is thought to have begun many years before the onset of symptoms. Therefore, we first set out to investigate the underlying changes in healthy control APOE genotypes  (i.e. E2/E2, E3/E3, and E4/E4) to determine whether underlying isoform specific differences exist in non-demented patients that could be prodromal to and contribute to AD pathogenesis. As E3/E3 is the most common genotype, we used the control E3/E3 cases as the normal (reference) phenotype in our comparisons. Moreover the use of homozygote genotypes ensured a cleaner analyses of the three different alleles/isoforms in isolation without the confounding influence in heterozygote genotypes. It is worthy of note that the mean age of E2/E2 patients we analyzed was 71yrs, while E3/E3 were slightly older, approximately 83yrs. Due to the scarcity of aged healthy control non-demented E4/E4 patients, we were only able to obtain samples from younger cohorts with a mean age of 45 yrs, thus this age bias has to be factored into the discussion of our results as a limitation of this study. The clinical background of the E2/E2 and E3/E3 controls demonstrated similar MMSE scores (28–29), low Braak staging (II) and plaque score (1–3). Due to the relatively young mean age of E4/E4 controls (most died as a result of natural causes unrelating to brain disorders) there was no detailed neuropathological examination conducted, thus we do not have information on their cognitive status or tau/amyloid pathology scores. 

We observed 217 significantly regulated proteins in E2/E2 vs E3/E3 controls and Ingenuity pathway analyses identified over 40 pathways. Notable changes included, EIF2 signaling, oxidative phosphorylation, mitochondrial dysfunction, phagosome maturation, chemokine and sirtuin signaling and DNA repair mechanisms. Of the  40 pathways significantly altered, 15 were unique to the E2/E2 vs E3/E3 comparison alone, with the most significant of these being mitochondrial dysfunction and Chemokine signaling. Thus indicating that the E2 allele/isoform may have specific roles in contributing towards aspects of energy bioenergetics and immune cell trafficking in the aged  brain. These proteomic changes appeared to involve a variety of different cell types, as expression levels of some significant proteins were found to be specific to smooth muscle cells, pericytes, astrocytes and endothelial cells.

Despite the relatively young age of the APOE4/4 cases, a host of molecular abnormalities were observed in their cerebrovessels when compared to the older E3/E3 cases. Firstly, 260 significant proteins were identified from this comparison, and 25 modulated pathways, most notably EIF2, sirtuin, eIF4/70S6K and mTOR signaling, phagosome maturation, remodeling of adherent junction and DNA repair mechanisms. Of the 25 pathways identified, two were unique to only the E4/E4 vs E3/E3 group (i.e. Granzyme A and semaphorin signaling). With respect to the cerebrovascular cells playing a role in these changes, we saw significant changes in cell specific markers related to endothelial cells and astrocytes.

Due to the age disparity in the E4/4 vs E3/3 groups and the possibility that the isoform specific effects we observed may be driven in part by differences in age, we also compared differences between E3/4 vs E3/3 groups, which were more closely matched with age. We still observed fifty nine significant proteins from this comparison, and seven modulated pathways, the notable ones being EIF2 signaling, TCA cycle, Calcium transport, HIF $\alpha$ and GM-CSF signaling. Given that EIF2 signaling was the top significantly modulated pathway between E4/E4 vs E3/E3 and E3/E4 vs E3/E3 cases, it suggests that APOE4 genotype may play a prominent role in regulating this molecular signaling pathway essential for protein translation.

Together, our findings reveal that underlying APOE genotype-specific proteomic differences in non-demented controls exists and may explain the early changes to cerebrovessels and the subsequent propensity for developing AD particularly in APOE4 carriers. On the other hand, the E2 specific effects we observed could also explain the association of this allele with an increased risk for CAA in the elderly, development of cerebral microhemorrhages in CAA patients, and an increased likelihood for cerebral ischemia in the elderly (independent of AD/amyloid pathology) [64–67]. Notably, the E2 genotype is also commonly attributed to hyperlipidemia and cardiovascular issues in the general population [68, 69]. Paradoxically, APOE2 also reduces the risk for CAA in AD patients, and protects against AD-related pathology compared to other APOE variants, as evidenced by the low frequency of E2/E2 AD cases in the general population [70, 71]. How these underlying cerebrovascular changes influences AD pathobiology remains elusive. Perhaps a limitation of this study is that we have not captured the extravascular related effects of E2 which may explain its protective role in mitigating AD pathogenesis. Further studies need to be explored in this genotype to identify the specific mechanisms driving their reduced  risk for developing AD.

### APOE specific effects on cerebrovascular proteome in AD

We next interrogated the interaction between APOE genotype and disease (i.e. AD diagnosis) from our cerebrovascular proteomic analyses. The demographics of the AD patients we used in this study revealed that all genotypes consisted of mixed gender, and all cases were octogenarians with a mean age between 84 and 89 yrs, except the E3/E4 cases which were the youngest with a mean age of 78yrs. As expected, the E4/E4 cases showed the most aggressive clinicopathological scores, with the worst scores for Tau, amyloid, CAA and white matter pathology, and the smallest brain weight on average compared to all other genotypes, while the E2/3 cases on the other hand demonstrated the least aggressive clinicopathological scores.

Our proteomic analyses of E4 carriers revealed 192 and 189 significantly regulated proteins that were unique to the interaction between disease and genotype, E3/E4 or E4/E4 respectively. Ingenuity pathway analyses identified, 24 and 14 pathways between disease and genotype interaction, E3/E4 or E4/E4 respectively. EIF2, eIF4 and 70S6K, and mTOR signaling were amongst the top significantly altered pathways from the disease and APOE (E3/E4 or E4/E4) genotype interactions, signifying that the APOE4 allele influences the molecular mechanisms driving protein translation/synthesis, metabolic pathways,  and UPR/ER stress and autophagy. Because we observed similar biological functions altered  between the E4/E4 vs E3/E3 controls, it is likely E4 genotype drives early cerebrovascular abnormalities in the aging process, which increases the vulnerability of the cerebrovasculature to further damage in AD pathogenesis. To corroborate our findings, previous studies have suggested that EIF2 $\alpha$ and mTOR pathways are major players in the APOE4 mediated cellular effects, and have been explored as therapeutic targets in mouse models of AD [72–76].

Of the total number of significantly altered pathways identified, 6 out of 14 were unique to only the disease and E4/E4 interactions, while 17 out of 24 were unique to the disease and E3/E4 interaction. Some of the Top pathways unique to the E4/E4 groups include Tight junction signaling, neucleobases (pyramidine) synthesis, and Integrin linked kinase signaling, and from the E3/E4 groups, sirtuin signaling pathway, remodeling of adherene junctions and Telemore extension.

With respect to the cerebrovascular cells playing a role in these changes, we saw significant changes in protein markers specific to smooth muscle cells, endothelial cells and astrocytes with disease and (E3/E4) genotype interaction, while only endothelial cells and astrocyte specific markers were identified from the E4/E4 groups.

Together our findings implicate a specific prominent role for the APOE4 allele in molecular mechanisms such as protein translation/synthesis, cell to cell contact signaling, BBB integrity, nucleic acid synthesis, cellular survival and metabolism, and ER stress/autophagy in cerebrovascular cells. Given that the E4 carriers showed the worst clinicopathological phenotypes that positively correlated with the extent of cerebrovascular abnormalities, we consider that these molecular mechanisms identified herein may provide tractable cerebrovascular specific targets for disease modifying strategies in AD, particularly for subjects carrying the high risk APOE4 genotype.

In contrast, our proteomic analyses of E3/E3 and E2/E3 carriers revealed 41 and 102 significantly regulated proteins that were unique to the interaction between disease and these respective APOE genotype interactions. Ingenuity pathway analyses identified 16 pathways from the disease and E3/E3 interaction and 5 pathways from the disease and E2/E3 interaction. Noted pathways modulated in the E3/E3 cohort  include EIF2, mTOR, eIF4 and p70S6K signaling, phagosome maturation, glutamine synthesis, CXCR4 signaling and aldosterone signaling. While Spliceosomal cycle, DNA methylation and transcriptional repression signaling, EIF2 signaling, DNA base excision repair pathway and DNA double strand break repair signaling were the only five pathways altered in the E2/E3 cohort.

Eleven out of 16 pathways were unique to the disease and E3/E3 genotype interaction, while 1 out of 5 was unique to the disease and E2/E3 genotype interaction. The top two pathways unique to the E3/E3 cohorts  were CXCR4 and aldosterone signaling, while DNA methylation and transcriptional repression signaling was the only pathway unique to E2/E3.

With respect to the cerebrovascular cells potentially influencing these proteomic changes, we saw significant changes in cell markers specific to only astrocytes from E2/E3 and E3/E3 AD cases vs controls, suggesting that E2 and E3 allele may play less prominent roles  on endothelial and mural cell pathobiology in AD compared to the E4 allele. Despite the unfavorable cerebrovascular changes observed in non-demented E2 allele carriers, E2/3 AD cases did not exhibit an augmented cerebrovascular phenotype in AD compared to the other APOE variants; their unique AD-related phenotype was typified by alterations in nucleic acid synthesis, transcriptional gene pathways and protein translation. Given that the molecular events in cerebrovessels differ significantly in the APOE2 genotype compared to other variants, targeting these distinct pathways in AD, may provide a unique strategy for combating the specific cerebrovascular changes in those carrying this alleles.

To our knowledge no studies have directly investigated the influence of APOE genotype on proteomic changes in cerebrovascular tissue from AD brains. The most relevant study we found used a comparative unbiased mass spectrometry-based method to interrogate post-mortem brain cortical tissues from pathologically-defined AD cases from E2/E3, E3/E3 and E4/E4 genotypes compared to control E3/E3 cases [77]. Using a weighted co-expression network analysis method, the authors identified 33 modules of co-expressed proteins, 12 of which were significantly different by APOE genotype in AD. The top modules impacted by E4/E4 genotype included mitochondrial function, synaptic transmission, inflammatory response, and a trend for protein translation, corroborating some of our findings in the E3/E4 and E4/E4 genotypes. Unlike our findings, their deconvolution cell type analyses showed a lack of effect on specific phenotypic cell type markers observed in the AD cases on the E3/E3 and E4/E4 background when compared to control E3/E3 cases. This was possibly due to the lack of appropriate controls for each genotype in the study design. The authors, however, did note that E2 allele carriers suppressed homeostatic and disease-associated AD phenotypic cell type marker changes in astrocytes, microglia, and endothelia, pointing to a possible neuroprotective feature of the E2 allele [77].

The precise mechanisms driving APOE genotype specific effects on neurological dysfunction has been largely discussed in the literature. Some of the earliest clues linking APOE with vascular degeneration in AD involved studies demonstrating a link between APOE-ε4 and increased amyloid deposition around blood vessels [38, 78–80]. APOE is thought to play a role in regulating the metabolism and perivascular drainage of Aβ from extracellular fluids in the brain. Intracerebral injection of Aβ into the brain of mice results in co-localization with APOE along basement membrane drainage pathways in the walls of cerebral arteries [81]. This clearance route is likely deficient in APOE4 carriers, as shown by the co-localization of APOE and non-fibrillar Aβ in the perivascular space and neuropil surrounding cerebral arteries of human AD brains [82] and also preclinical transgenic models of amyloidosis on the human APOE background [81, 83–86]. This event is possibly driven by APOE early in the sequelae of AD, as APOE has been found to accumulate in the early stages of senile plaque formation, preceding Aβ deposition in meningeal vessels in amyloid angiopathy [87]. Further support of this role on amyloid clearance, have also been corroborated by studies showing that APOE4/Aβ complexes form a weaker bond formation with basement membrane proteins along cerebral arteries compared to APOE3/Aβ complexes [86]. Other studies have also shown a direct impact of APOE on BBB permeability by altering transport mechanisms at the level of endothelial cells [88, 89]. For example, studies in APOE knock-in mice have revealed an age-related (12Mos > 6Mos) and isoform specific (E4 > E3) increase in the mRNA levels of influx and efflux Aβ transporters [89]. We also previously showed that APOE4 isoform can influence MMP9 induced shedding of an efflux transporter (LRP-1) of Aβ and its subsequent BBB transport of APOE-Aβ complexes [59]. The consequences of APOE on Aβ clearance and the subsequent increase in toxic amyloid deposits along the walls of the vasculature could be a plausible mechanism  driving the toxicity of cerebrovessels in the pathogenesis of AD, leading to the molecular abnormalities we observed in this study.

Other studies have provided an alternate narrative of these events that implicates a direct role for APOE on cerebrovessel damage independent of its effects on Aβ. Early clues to support this hypothesis originated from seminal work showing a significant correlation between APOE allele and cerebral small-vascular diseases (CSVD) typified by white matter hyperintensities, dilated perivascular space, microbleeds and lacunae [40, 41, 67]. Severity of CSVD has been reported to correlate with the localization of APOE and plasma derived IgG proteins around CSVD altered cerebrovessel walls [40, 41]. Studies have also shown an association between APOE4 and atherosclerotic middle cerebral artery stenosis in cerebral ischemia [43], and a relation between coronary atherosclerosis and APOE4 genotypes in AD cases [44]. These effects have been suggested to be influenced in part by the impact of APOE4 allele on peripheral lipid profiles (i.e. higher total cholesterol, LDL cholesterol and apoliporotein B levels in the plasma) [69] and the innate immune response, and their combined indirect influence on cerebrovessels.

Other investigators have speculated that changes in APOE levels in the brain may drive AD pathogenesis [90]. We therefore interrogated the levels of APOE in cerebrovessels of control and AD cases, and observed no significant change across genotype in non-demented cases, but unexpectedly saw a significant increase in E4/E4 AD cases compared to controls. Although some studies have reported an inverse correlation between brain APOE expression and Aβ load [90, 91], a closer look at the literature suggests inconsistent results when measuring APOE levels in the brain as a function of APOE genotype [92–98]. In our study, the E4/E4 AD and control cases were poorly age-matched, which could be a driving factor in the differences observed. Larger cohorts of age-matched cases will be needed to confirm this finding in E4 carriers, and to demonstrate whether this is a unique feature observed in the cerebrovessels of E4 carriers. Further studies will also be needed to investigate the cellular origin and upstream regulators  driving these changes, how APOE expression levels impacts on cerebrovascular cell dysfunction, and whether this event occurs prior to the onset of Aβ deposition.

More recent work has confirmed the link between APOE4 and the accelerated breakdown of the BBB in vulnerable brain regions such as the medial temporal lobe in unimpaired, and to a greater extent in cognitively impaired patients, independent of amyloid and tau pathology [46, 47, 49]. This has also been confirmed in human APOE4 expressing mice showing early vascular changes (such as BBB damage, influx of neurotoxic serum proteins, early microvascular and cerebral blood flow reductions [42]) that precede the early onset of neuronal dysfunction and subsequent neurodegeneration. This effect is thought to be mediated by the influence of APOE on pericyte dysfunction. Early increase in CSF levels of soluble PDGFRβ, degeneration of pericytes, and their coverage of brain capillaries have been shown in human and transgenic AD models on the APOE4 background [42, 47, 99, 100]. Damage to pericytes could impact on the vasoconstrictive properties of cerebral microvessels leading to diminished blood flow, impaired perivascular drainage, and cognitive decline [101–103]. These effects on BBB integrity and pericyte dysfunction have been shown to be mediated by the increase in Cyclophilin (Cyp) production, which activates NF-κB induced upregulation of MM9, involved in degrading capillary basement membrane and tight junction proteins [42, 47, 99]. Consistent  with   our study, damage to BBB integrity, aberrant regulation of protein translation, deficits in cellular survival and metabolism, and autophagy were notably altered in APOE4 cases with AD. Interfering with these pathways and those identified in our studies, using pharmacological inhibitors, short interfering RNA, or genetic deletion may provide an avenue to reverse weakened cerebrovascular integrity  and BBB dysfunction in AD.

Another possible explanation of the influence of APOE on cerebrovascular dysfunction could be through its indirect effect on inflammatory processes in glial cells. Previous work has shown that glial cells play an essential role in driving APOE4 mediated neurodegeneration in preclinical models of AD-like pathology [104–107]. APOE4 has also been shown to drive widespread molecular and cellular alterations associated with AD pathogenesis in isogenic APOE carrying glial cells derived from inducible pluripotent stem cells (iPSC) from AD patients [108]. Our cerebrovascular tissue showed moderate expression of glial markers, therefore some of the APOE specific effects we observed could be driving in part by changes in these cell types, which are the main producers of APOE in the brain [33]. APOE4 genotype has been demonstrated to influence the phenotypic change of astrocytes into the A1 disease phenotype, and also convert microglia into neurotoxic entities that produce proinflammatory cytokines [105, 108–112]. In AD, glial cell inflammation could therefore serve as critical determinants driving the immune response that contributes to the damage of cerebrovessels. In this study, we saw several astrocyte cell specific markers (e.g. APOE, GFAP, S100B, AQ4) that were significantly altered in control vs AD cases from all APOE backgrounds. This correlated with changes in serum derived proteins such as thrombin factors, increased expression of integrins, adhesion molecules and chemokines between AD  cases and controls, in E3 and E4 carriers. Increased expression of adhesion molecules and integrins can make vessels more adhesive, whereby immune cells such as macrophages/foamy cells and neutrophils could infiltrate the brain environment, and also clog up vessel walls leading to a reduction in blood flow and inflammatory damage to cerebrovessels [113].

## Conclusion

Our work has provided a snapshot of the molecular changes that exists in the cerebrovasculature of AD and non-demented brains from different APOE genotypes. One limitation of this study design is our inability to determine whether these disrupted cellular functions precede or are a direct consequence of amyloid or tau pathology in AD. A larger sample size (well matched for all genotypes, sex and age) and longitudinal study design focusing on the neuropathological staging of disease will be needed to elucidate the specific effect of APOE on the sequelae of cerebrovascular dysfunction in AD pathobiology. Future studies could also incorporate phosphoproteomic approaches to identify phosphorylation status of EIF2 and mTOR specific pathways that were highly enriched in our proteomic datasets. The use of single cell approaches with tools such as FACS, single cell transcriptomics (scRNAseq) and RNAscope may also be invaluable, and will complement our total proteomic method which lacked the ability to detect the specific cellular origins of each differentially expressed proteins.

Together, our findings in this study have provided novel insights into the impact of APOE genotype on the brain vasculature in normal and AD conditions. The cerebrovasculature is crucial in maintaining brain homeostasis, neurovascular coupling, and supplying oxygen and glucose to the highly active cells of the brain. We consider that the APOE dependent changes identified herein may generate novel targets for developing therapies that protect against damage to the cerebrovasculature and mitigate the risk for AD conferred by APOE genotype.

## Supplementary Information

**Additional file 1: Table S1.** List of significantly regulated proteins in the cerebrovasculature of the inferior frontal gyrus in healthy homozygote control cases from APOE2/E2 vs APOE3/E3 genotypes. Data are expressed as the negative Log10 of the p value (green horizontal bars—significance cut off set at > 1.3), and the Log2 fold change between control cases from APOE2/E2 vs APOE3/E3 genotypes. Heat map indicates downregulated (Red box) or upregulated (Blue box) proteins. Statistical analyses was performed using two way ANOVA after logarithmic transformation.

**Additional file 2: Table S2.**List of significantly regulated proteins in the cerebrovasculature of the inferior frontal gyrus in healthy homozygote control cases from APOE4/E4 vs APOE3/E3 genotypes. Data are expressed as the negative Log10 of the p value (green horizontal bars—significance cut off set at > 1.3), and the Log2 fold change between control cases from APOE4/E4 vs APOE3/E3 genotypes Heat map indicates downregulated (Red box) or upregulated (Blue box) proteins. Statistical analyses was performed using two way ANOVA after logarithmic transformation.

**Additional file 3: Table S3.** List of significantly regulated proteins in the cerebrovasculature of the inferior frontal gyrus in healthy homozygote control cases from APOE3/E4 vs APOE3/E3 genotypes. Data are expressed as the negative Log10 of the p value (green horizontal bars—significance cut off set at > 1.3), and the Log2 fold change between control cases from APOE3/E4 vs APOE3/E3 genotypes Heat map indicates downregulated (Red box) or upregulated (Blue box) proteins. Statistical analyses was performed using two way ANOVA after logarithmic transformation.

**Additional file 4: Table S4.** List of significantly regulated proteins in the cerebrovasculature of the inferior frontal gyrus in Alzheimer's disease and matched control cases from E2/E3 genotypes. Data are expressed as the negative Log10 of the p value (green horizontal bars—significance cut off set at > 1.3), and the Log2 fold change between AD and matched control cases from E2/E3 genotypes. Heat map indicates downregulated (Red box) or upregulated (Blue box) proteins. Statistical analyses was performed using two way ANOVA after logarithmic transformation.

**Additional file 5: Table S5.** List of significantly regulated proteins in the cerebrovasculature of the inferior frontal gyrus in Alzheimer's disease and matched control cases from E3/E3 genotypes. Data are expressed as the negative Log10 of the p value (green horizontal bars—significance cut off set at > 1.3), and the Log2 fold change between AD and matched control cases from E3/E3 genotypes. Heat map indicates downregulated (Red box) or upregulated (Blue box) proteins. Statistical analyses was performed using two way ANOVA after logarithmic transformation.

**Additional file 6: Table S6.** List of significantly regulated proteins in the cerebrovasculature of the inferior frontal gyrus in Alzheimer's disease and matched control cases from E3/E4 genotypes. Data are expressed as the negative Log10 of the p value (green horizontal bars—significance cut off set at > 1.3), and the Log2 fold change between AD and matched control cases from E3/E4 genotypes. Heat map indicates downregulated (Red box) or upregulated (Blue box) proteins. Statistical analyses was performed using two way ANOVA after logarithmic transformation.

**Additional file 7: Table S7.** List of significantly regulated proteins in the cerebrovasculature of the inferior frontal gyrus in Alzheimer's disease and matched control cases from E4/E4 genotypes. Data are expressed as the negative Log10 of the p value (green horizontal bars—significance cut off set at > 1.3), and the Log2 fold change between AD and matched control cases from E4/E4 genotypes. Heat map indicates downregulated (Red box) or upregulated (Blue box) proteins. Statistical analyses was performed using two way ANOVA after logarithmic transformation.

## Data Availability

Our mass spectrometry data has been deposited into the ProteomeXchange Consortium via the PRIDE partner repository. Our datasets can be located with the unique identifier – PXD023340.
