# Supplementary figures and images for: APOE genotype dependent molecular abnormalities in the cerebrovasculature of Alzheimer’s disease and age-matched non-demented brains
Source: Mol Brain. 2021 Jul 8;14:110. doi: 10.1186/s13041-021-00803-9 (PMC8268468; doi:10.1186/s13041-021-00803-9)

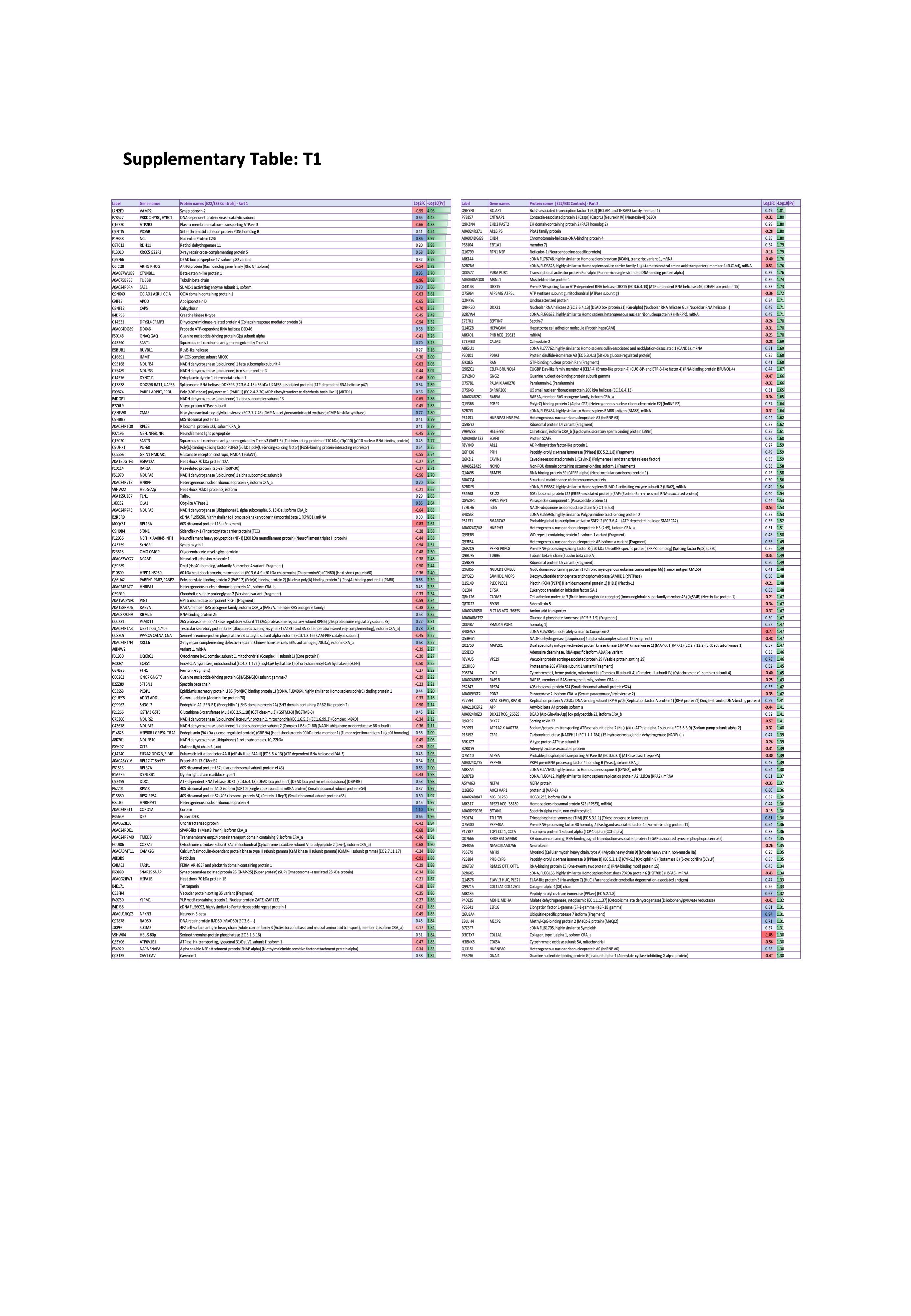

Supplement: Supplementary file 1 — Additional file 1: Table S1. List of significantly regulated proteins in the cerebrovasculature of the inferior frontal gyrus in healthy homozygote control cases from APOE2/E2 vs APOE3/E3 genotypes. Data are expressed as the negative Log10 of the p value (green horizontal bars—significance cut off set at > 1.3), and the Log2 fold change between control cases from APOE2/E2 vs APOE3/E3 genotypes. Heat map indicates downregulated (Red box) or upregulated (Blue box) proteins. Statistical analyses was performed using two way ANOVA after logarithmic transformation. [file 13041_2021_803_MOESM1_ESM.jpeg]

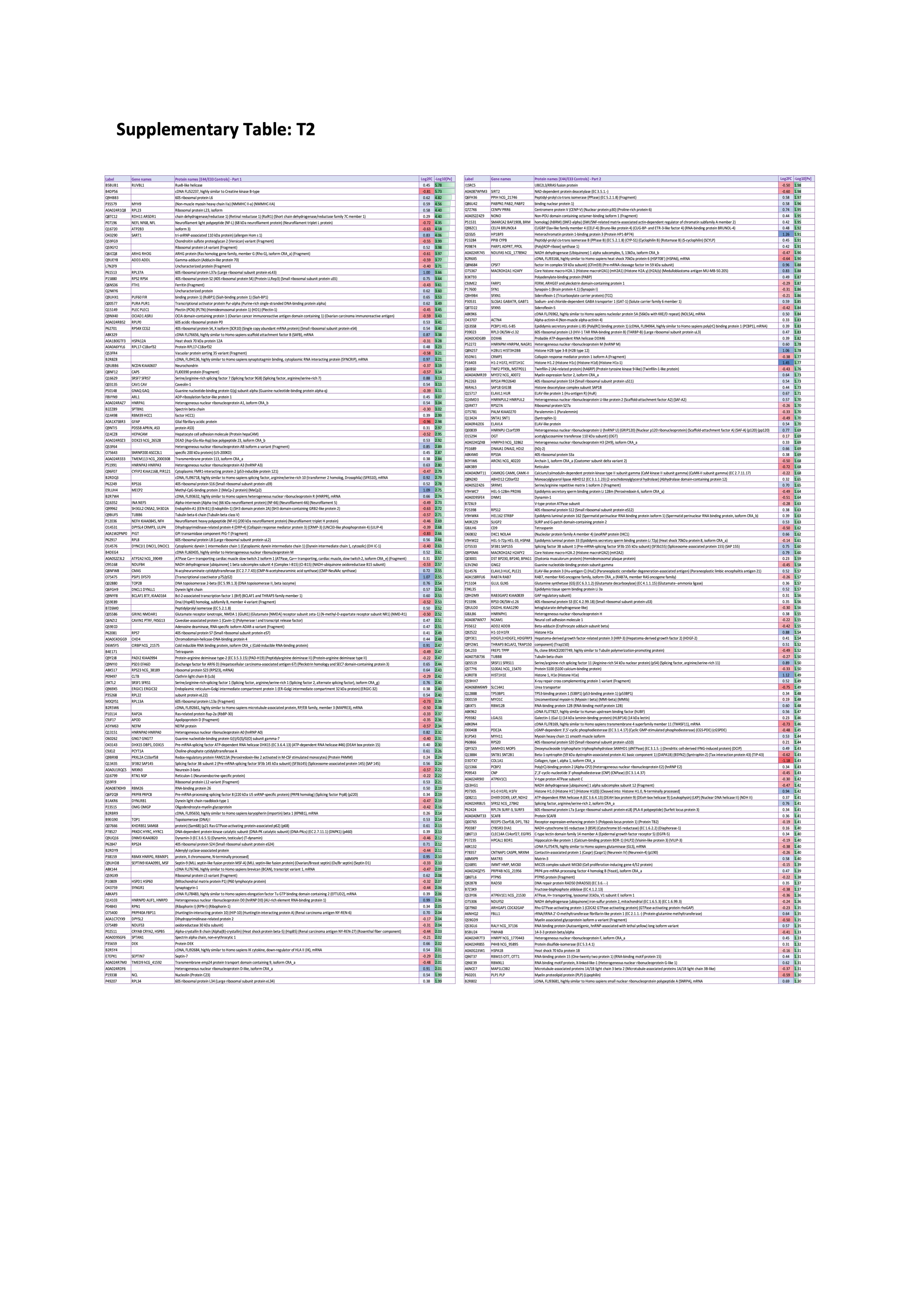

Supplement: Supplementary file 2 — Additional file 2: Table S2.List of significantly regulated proteins in the cerebrovasculature of the inferior frontal gyrus in healthy homozygote control cases from APOE4/E4 vs APOE3/E3 genotypes. Data are expressed as the negative Log10 of the p value (green horizontal bars—significance cut off set at > 1.3), and the Log2 fold change between control cases from APOE4/E4 vs APOE3/E3 genotypes Heat map indicates downregulated (Red box) or upregulated (Blue box) proteins. Statistical analyses was performed using two way ANOVA after logarithmic transformation. [file 13041_2021_803_MOESM2_ESM.jpeg]

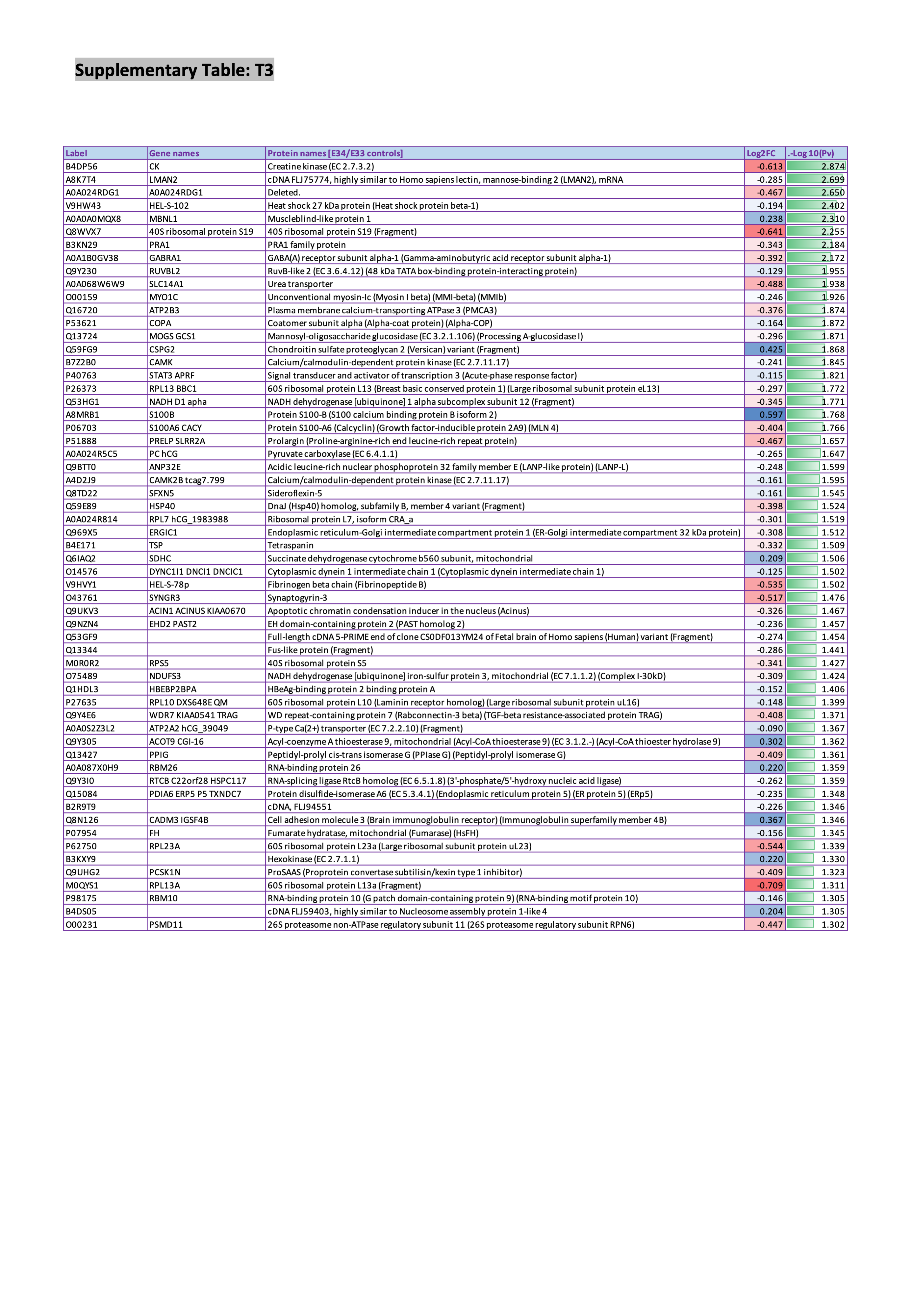

Supplement: Supplementary file 3 — Additional file 3: Table S3. List of significantly regulated proteins in the cerebrovasculature of the inferior frontal gyrus in healthy homozygote control cases from APOE3/E4 vs APOE3/E3 genotypes. Data are expressed as the negative Log10 of the p value (green horizontal bars—significance cut off set at > 1.3), and the Log2 fold change between control cases from APOE3/E4 vs APOE3/E3 genotypes Heat map indicates downregulated (Red box) or upregulated (Blue box) proteins. Statistical analyses was performed using two way ANOVA after logarithmic transformation. [file 13041_2021_803_MOESM3_ESM.tiff]

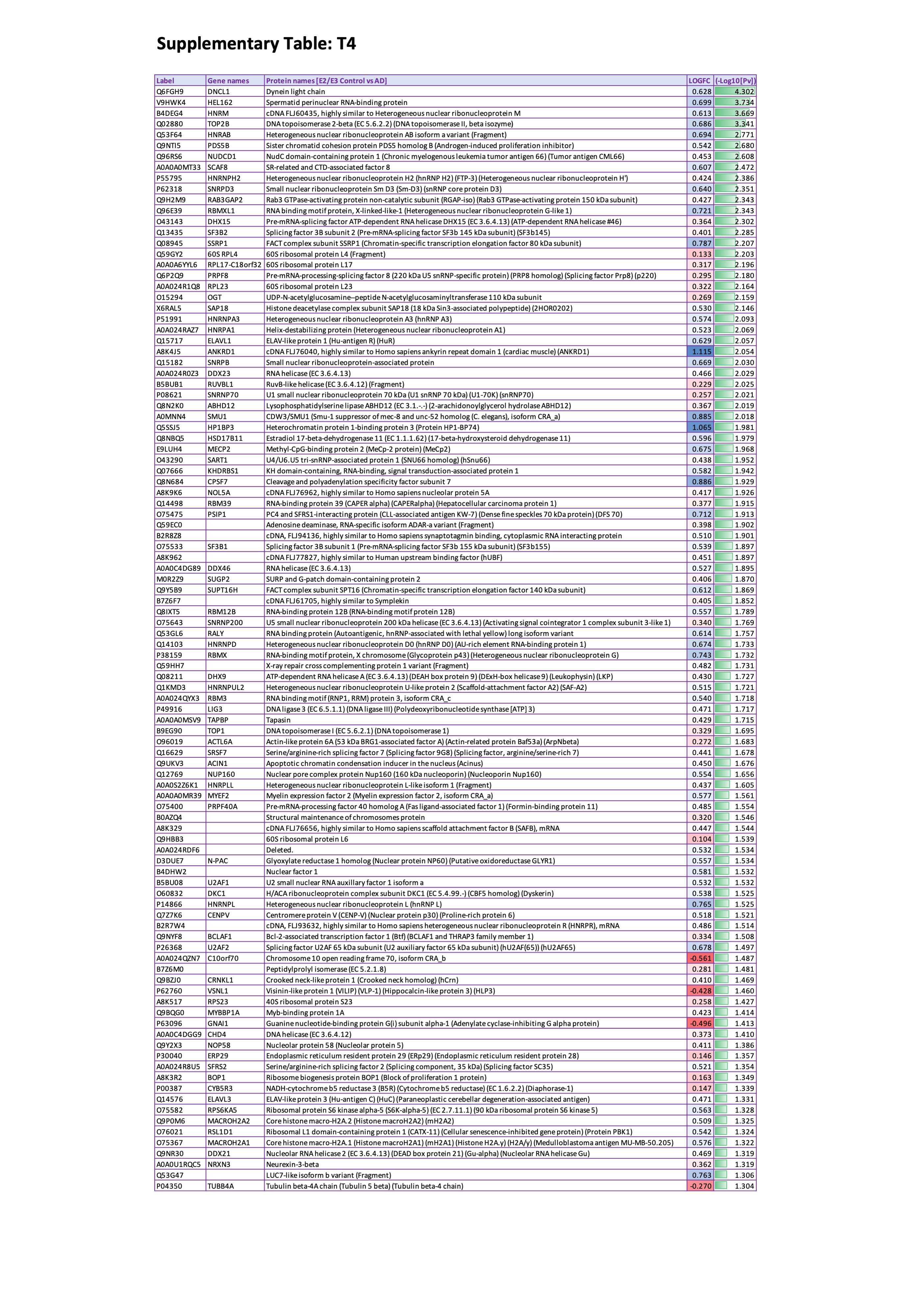

Supplement: Supplementary file 4 — Additional file 4: Table S4. List of significantly regulated proteins in the cerebrovasculature of the inferior frontal gyrus in Alzheimer's disease and matched control cases from E2/E3 genotypes. Data are expressed as the negative Log10 of the p value (green horizontal bars—significance cut off set at > 1.3), and the Log2 fold change between AD and matched control cases from E2/E3 genotypes. Heat map indicates downregulated (Red box) or upregulated (Blue box) proteins. Statistical analyses was performed using two way ANOVA after logarithmic transformation. [file 13041_2021_803_MOESM4_ESM.jpeg]

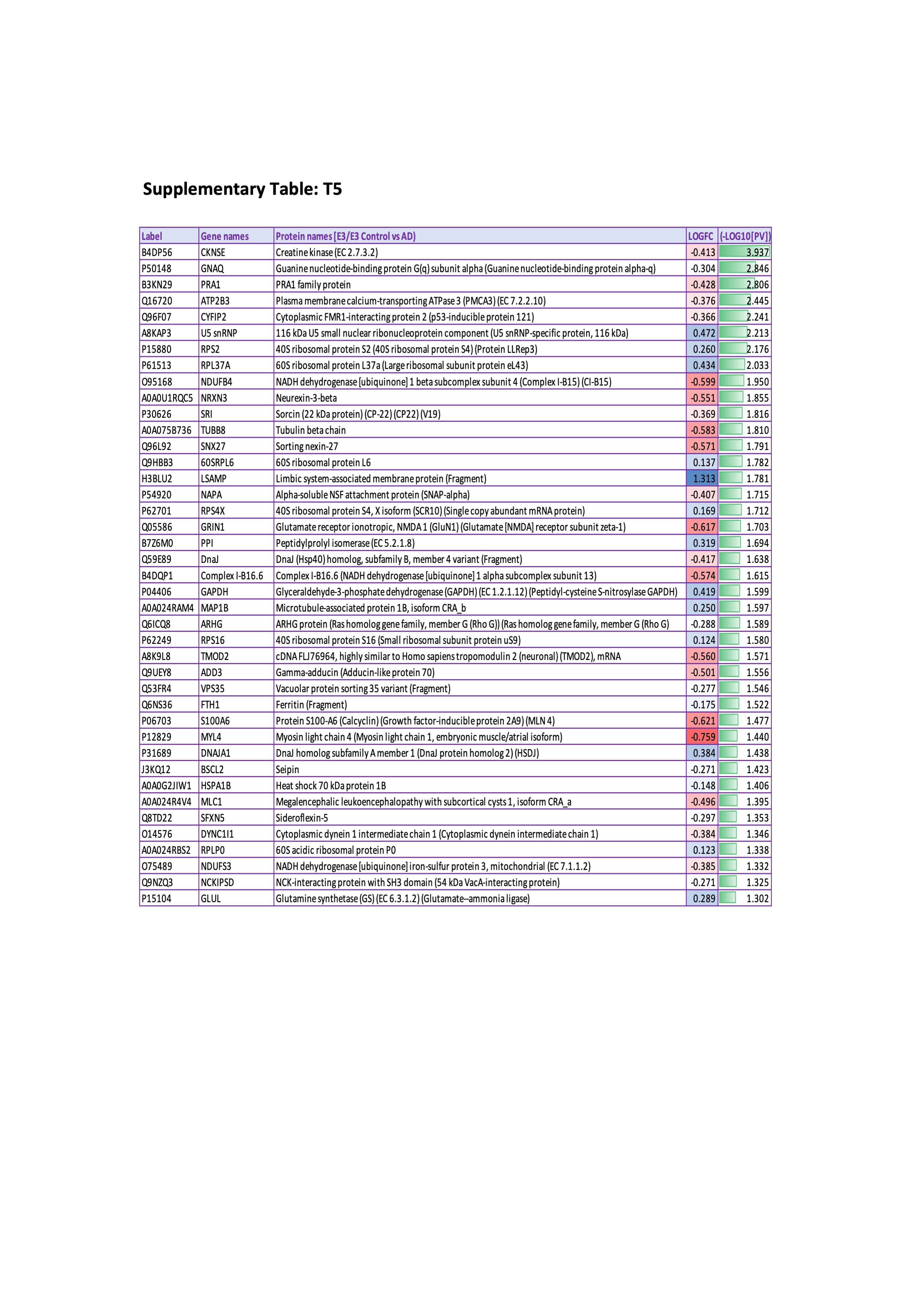

Supplement: Supplementary file 5 — Additional file 5: Table S5. List of significantly regulated proteins in the cerebrovasculature of the inferior frontal gyrus in Alzheimer's disease and matched control cases from E3/E3 genotypes. Data are expressed as the negative Log10 of the p value (green horizontal bars—significance cut off set at > 1.3), and the Log2 fold change between AD and matched control cases from E3/E3 genotypes. Heat map indicates downregulated (Red box) or upregulated (Blue box) proteins. Statistical analyses was performed using two way ANOVA after logarithmic transformation. [file 13041_2021_803_MOESM5_ESM.jpeg]

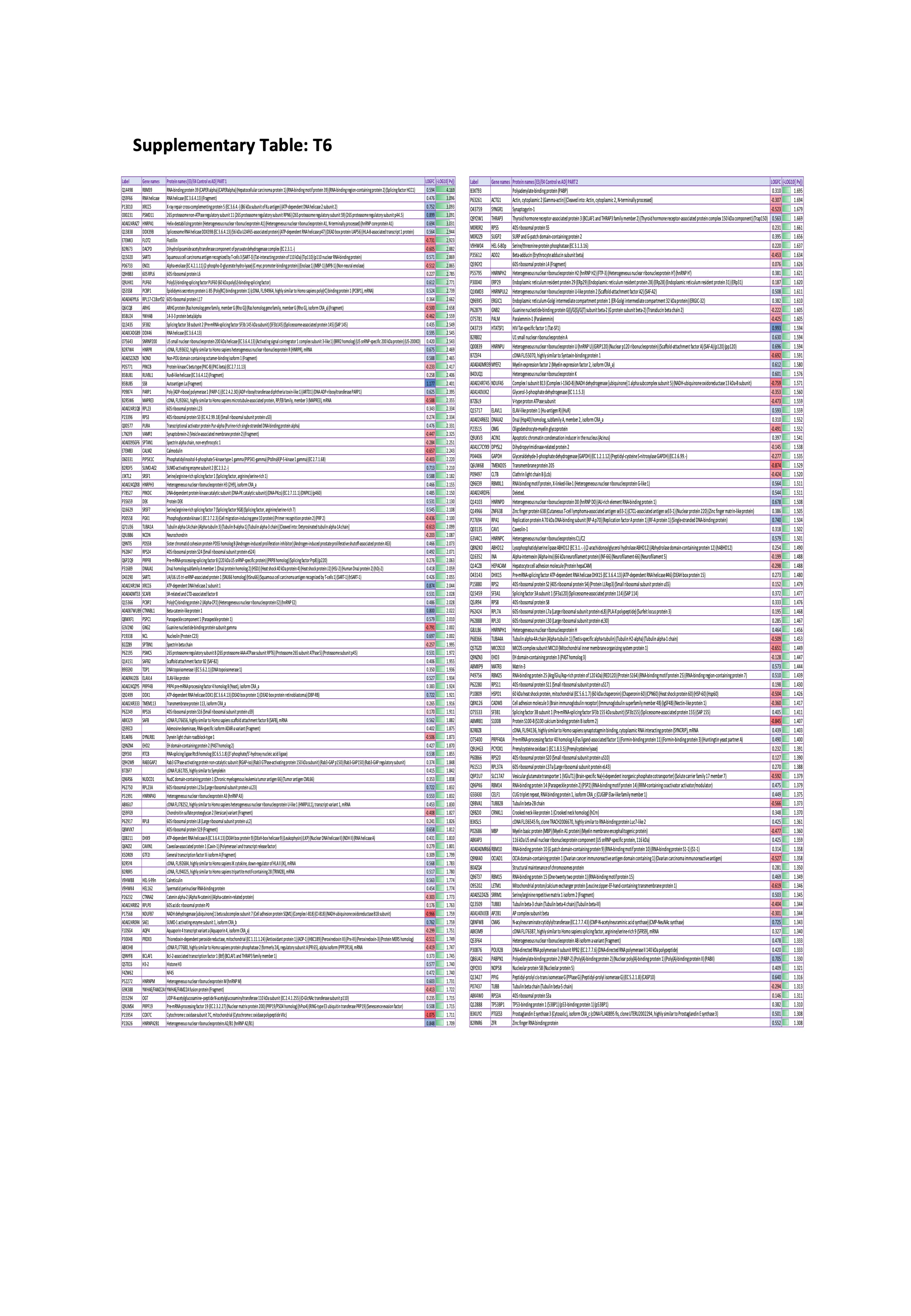

Supplement: Supplementary file 6 — Additional file 6: Table S6. List of significantly regulated proteins in the cerebrovasculature of the inferior frontal gyrus in Alzheimer's disease and matched control cases from E3/E4 genotypes. Data are expressed as the negative Log10 of the p value (green horizontal bars—significance cut off set at > 1.3), and the Log2 fold change between AD and matched control cases from E3/E4 genotypes. Heat map indicates downregulated (Red box) or upregulated (Blue box) proteins. Statistical analyses was performed using two way ANOVA after logarithmic transformation. [file 13041_2021_803_MOESM6_ESM.jpeg]

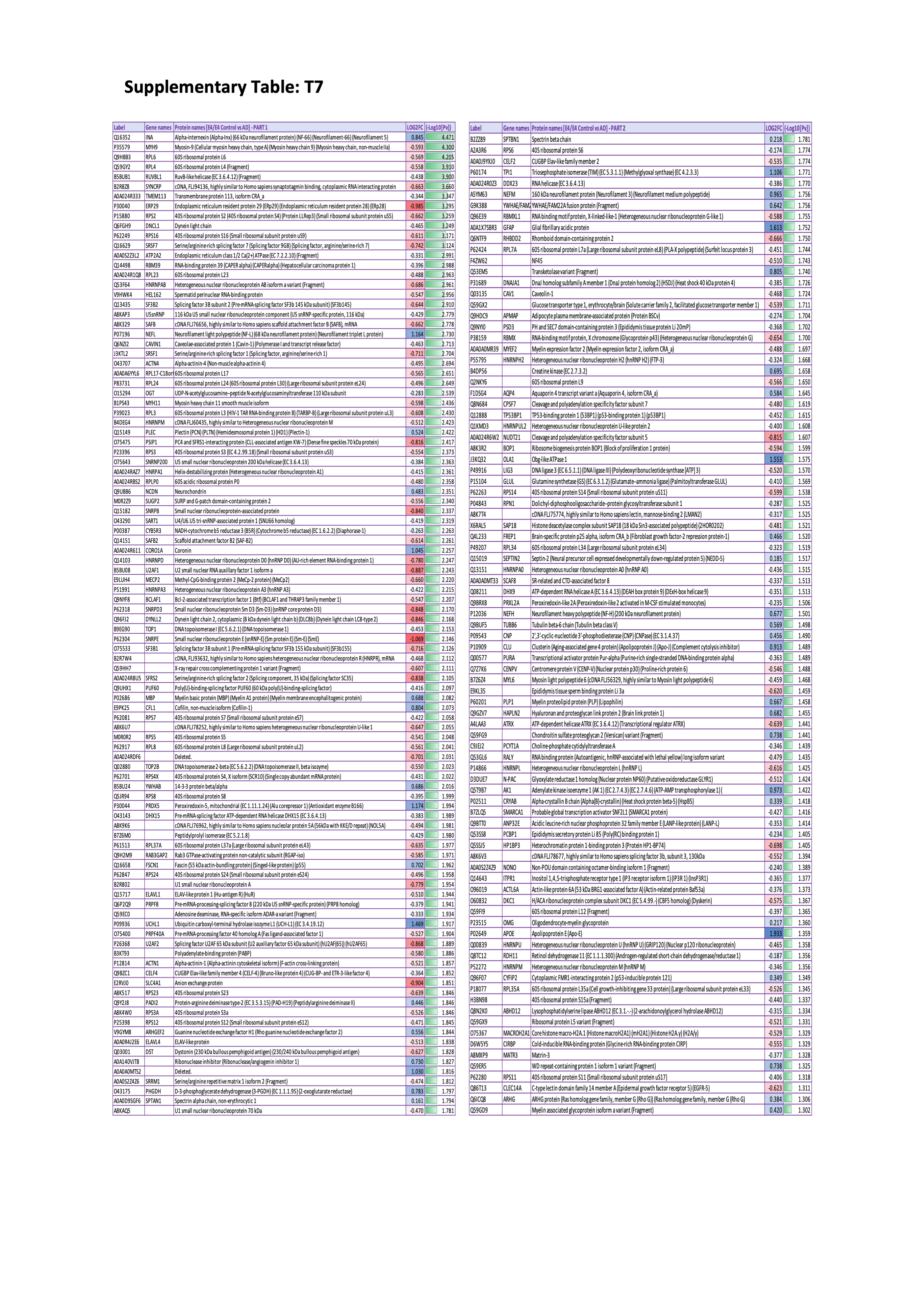

Supplement: Supplementary file 7 — Additional file 7: Table S7. List of significantly regulated proteins in the cerebrovasculature of the inferior frontal gyrus in Alzheimer's disease and matched control cases from E4/E4 genotypes. Data are expressed as the negative Log10 of the p value (green horizontal bars—significance cut off set at > 1.3), and the Log2 fold change between AD and matched control cases from E4/E4 genotypes. Heat map indicates downregulated (Red box) or upregulated (Blue box) proteins. Statistical analyses was performed using two way ANOVA after logarithmic transformation. [file 13041_2021_803_MOESM7_ESM.jpeg]
